# Ghost mitochondria drive metastasis through adaptive GCN2/Akt therapeutic vulnerability

**DOI:** 10.1073/pnas.2115624119

**Published:** 2022-02-17

**Authors:** Jagadish C. Ghosh, Michela Perego, Ekta Agarwal, Irene Bertolini, Yuan Wang, Aaron R. Goldman, Hsin-Yao Tang, Andrew V. Kossenkov, Catherine J. Landis, Lucia R. Languino, Edward F. Plow, Annamaria Morotti, Luisa Ottobrini, Marco Locatelli, David W. Speicher, M. Cecilia Caino, Joel Cassel, Joseph M. Salvino, Marie E. Robert, Valentina Vaira, Dario C. Altieri

**Affiliations:** ^a^Prostate Cancer Discovery and Development Program, The Wistar Institute, Philadelphia, PA 19104;; ^b^Immunology, Microenvironment and Metastasis Program, The Wistar Institute, Philadelphia, PA 19104;; ^c^Proteomics and Metabolomics Shared Resource, The Wistar Institute, Philadelphia, PA 19104;; ^d^Bioinformatics Shared Resource, The Wistar Institute, Philadelphia, PA 19104;; ^e^Center for Systems and Computational Biology, The Wistar Institute, Philadelphia, PA 19104;; ^f^Department of Cancer Biology, Sidney Kimmel Cancer Center, Thomas Jefferson University, Philadelphia, PA 19107;; ^g^Department of Cardiovascular and Metabolic Sciences, Lerner Research Institute, Cleveland Clinic, Cleveland, OH 44195;; ^h^Division of Pathology, Fondazione IRCCS Ca' Granda Ospedale Maggiore Policlinico, Milan 20122, Italy;; ^i^Department of Pathophysiology and Transplantation, University of Milan, Milan 20122, Italy;; ^j^Division of Neurosurgery, Fondazione IRCCS Ca’ Granda Ospedale Maggiore Policlinico, Milan 20122, Italy;; ^k^Department of Pharmacology, University of Colorado School of Medicine, Aurora, CO 80045;; ^l^Molecular Screening and Protein Expression Shared Resource, The Wistar Institute, Philadelphia, PA 19104;; ^m^Molecular and Cellular Oncogenesis Program, The Wistar Institute, Philadelphia, PA 19104;; ^n^Department of Pathology, Yale University School of Medicine, New Haven, CT 06510

**Keywords:** mitochondria, cell motility, metastasis

## Abstract

Cancer metabolism, including in mitochondria, is a disease hallmark and therapeutic target, but its regulation is poorly understood. Here, we show that many human tumors have heterogeneous and often reduced levels of Mic60, or Mitofilin, an essential scaffold of mitochondrial structure. Despite a catastrophic collapse of mitochondrial integrity, loss of bioenergetics, and oxidative damage, tumors with Mic60 depletion slow down cell proliferation, evade cell death, and activate a nuclear gene expression program of innate immunity and cytokine/chemokine signaling. In turn, this induces epithelial-mesenchymal transition (EMT), activates tumor cell movements through exaggerated mitochondrial dynamics, and promotes metastatic dissemination in vivo. In a small-molecule drug screen, compensatory activation of stress response (GCN2) and survival (Akt) signaling maintains the viability of Mic60-low tumors and provides a selective therapeutic vulnerability. These data demonstrate that acutely damaged, “ghost” mitochondria drive tumor progression and expose an actionable therapeutic target in metastasis-prone cancers.

## Significance

Exploitation of mitochondrial functions promotes tumor traits, including metastasis, which is responsible for >90% of all cancer deaths. In this study, we investigated how mitochondrial fitness impacts tumor behavior. We found that acutely damaged, de-energized, and reactive oxygen species-producing mitochondria not only persist in cancer but are also key enablers of metastasis. These “ghost” mitochondria originate from the heterogeneous and often reduced expression of Mic60, an essential scaffold of organelle structure, in certain human cancers. The compensatory activation of gene expression programs as well as GCN2/Akt kinase signaling enables the survival of Mic60-low tumors but also provides a new therapeutic target in advanced and hard-to-treat malignancies.

The rewiring of metabolic pathways is a ubiquitous cancer trait that confers cellular plasticity, expands clonal heterogeneity, and enables disease progression (1). There is now a consensus that mitochondria are important for this process, titrating energy output, buffering oxidative stress, and controlling a host of cell death programs (2). In particular, exploitation of mitochondrial functions has been linked to metastatic competence (3, 4). This involves oxidative bioenergetics (5) and redox balance (6) but also deregulated mitochondrial dynamics (7), a process that controls the size, shape, and distribution of mitochondria and their trafficking to the cortical cytoskeleton, where they fuel pivotal steps of cell motility, such as membrane lamellipodia dynamics, turnover of focal adhesion (FA) and phosphorylation of signaling kinases (8).

However, the environment of tumor growth is highly unfavorable to mitochondrial fitness. Erratic oxygen concentrations, high levels of oxidative radicals (9), constantly changing metabolic needs (10), and vulnerabilities of the mitochondrial proteome (11) are all potent stimuli to disrupt mitochondrial integrity, shut off organelle functions, and activate cell death (12). Quality-control measures activated in these settings, in particular mitophagy (13), are designed to remove such subpar, “ghost” mitochondria and restore homeostasis. However, the role of these pathways in cancer is far from clear, and activation of mitophagy has been paradoxically linked to tumor progression (14) as well as treatment resistance (15).

An important regulator of mitochondrial integrity is Mic60, also called Mitofilin or inner membrane mitochondrial protein. Mic60 is an essential constituent of a MICOS complex (16) that maintains cristae architecture (17), organizes respiratory complexes (18), and ensures outer membrane biogenesis (19). Whether this pathway is important in cancer has not been determined, but there is evidence that Mic60 participates in mitochondrial fitness, including PINK1/Parkin-directed mitophagy (20) and mitochondrial dynamics (21).

In this study, we investigated how mitochondrial fitness may impact cancer traits and potentially expose therapeutic vulnerabilities in advanced disease.

## Results

### Mic60 Expression in Cancer.

**Fig. 1.**
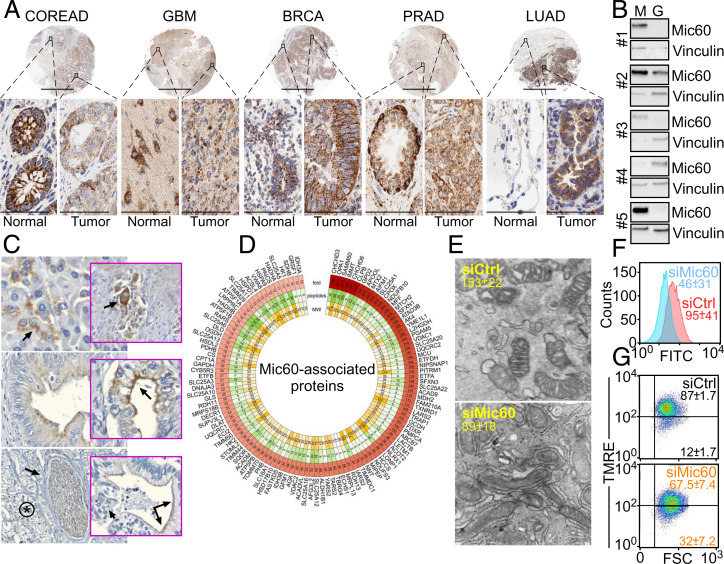
Mic60 expression in cancer. (*A*) A universal TMA was analyzed for differential expression of Mic60 in tumor vs. adjacent normal tissue by IHC. N, normal; T, tumor. Cases of COREAD (*n* = 6N, 8T), GBM (*n* = 5N, 8T), BRCA (*n* = 5N, 8T), PRAD (*n* = 5N, 5T), and LUAD (*n* = 6N, 8T) were examined. Representative images are shown. Scale bars, low magnification, 6 mm; high magnification, 60 μm (×40). (*B*) Patient-derived tissue samples (#1 to #5) of GBM (G) or disease-free margin (M) were analyzed by Western blotting. (*C*) Intratumoral heterogeneity of Mic60 expression in PDAC patients. *Top Left*, arrow, perinuclear expression in normal acinar cells (×200); *Top Right*, perinuclear to diffuse cytoplasmic staining in nerves, smooth muscle, and ganglion cells (arrow, ×200); *Middle Left*, apical cytoplasmic staining of high-grade pancreatic intraepithelial neoplasia (×400); *Middle Right*, faint perinuclear as well as bright apical staining (arrow) of well-differentiated PDAC (×400); *Bottom Left*, asterisk, absent stain in high-grade basaloid PDAC, arrow (×200); *Bottom Right*, transition between Mic60-positive well differentiated tumor (double arrows) and Mic60-negative high grade basaloid regions within the same tumor gland (single arrow) (×400). (*D*) Mic60 interactome identified in PC3 cells by mass spectrometry. The fold vs. IgG condition, number of detected peptides, and molecular weight (MW) are indicated. (*E*) PC3 cells transfected with control nontargeting siRNA (siCtrl) or Mic60-directed siRNA (siMic60) were analyzed by transmission electron microscopy, and cristae length (mean ± SD) was quantified (*n* = 25). *P* < 0.0001. (*F*) PC3 cells as in *E* were analyzed for mitochondrial outer membrane permeability by calcein staining and flow cytometry. Numbers correspond to mean fluorescence intensity (MFI; mean ± SD, *n* = 3). (*G*) PC3 cells as in *E* were analyzed for mitochondrial inner membrane potential by tetramethylrhodamine, ethyl ester (TMRE) staining and flow cytometry. MFI (mean ± SD) is shown (*n* = 3). *P* = 0.01.

To study the role of mitochondrial fitness in cancer, we focused on Mic60 as an essential scaffold of organelle integrity and function (18). Inspection of the Human Protein Atlas database showed that Mic60 expression was highly heterogeneous in cancer, as several tumor types had reduced, increased, or unchanged levels of Mic60 compared to normal tissues ( *SI Appendix*, Fig. S1*A* ). Immunohistochemical (IHC) staining of a universal tumor microarray (TMA; *n* = 5 to 8 cases per tumor type) gave similar results (Fig. 1*A*), where Mic60 expression was reduced in colorectal adenocarcinoma (COREAD) and glioblastoma (GBM), unchanged in breast (BRCA) and prostate adenocarcinoma (PRAD), or increased in lung adenocarcinoma (LUAD) compared to adjacent normal tissue ( *SI Appendix*, Fig. S1*B* ). Consistent with these results, Mic60 mRNA levels in The Cancer Genome Atlas (TCGA) database were reduced in GBM and COREAD but prominently upregulated in LUAD ( *SI Appendix*, Fig. S1*C* ). Other potential Mic60-low tumors in this analysis included malignancies of kidney, thyroid, head and neck, and soft tissue, whereas uterine and cervix cancer had higher Mic60 mRNA levels compared to normal tissues ( *SI Appendix*, Fig. S1*C* ). Although breast cancer showed increased Mic60 protein ( *SI Appendix*, Fig. S1*A* ) and mRNA ( *SI Appendix*, Fig. S1*C* ) in public databases, our TMA analysis did not reach statistical significance ( *SI Appendix*, Fig. S1*B* ). Heterogeneous Mic60 expression was also observed intratumorally. When analyzed in patient samples of pancreatic ductal adenocarcinoma (PDAC), Mic60 expression ranged from focal perinuclear distribution in normal pancreatic acinar cells to disordered, submembranous or linear staining in in situ and invasive neoplastic epithelium to absence in poorly differentiated (basaloid) carcinomas by IHC (Fig. 1*C*). Mechanistically, differentiation of patient-derived GBM neurospheres, a process associated with the modulation of stemness and proliferative potential (22), lowered Mic60 as well as HIF1α mRNA levels ( *SI Appendix*, Fig. S1*D* ).

### Mic60-Dependent Mitochondrial Integrity in Cancer.

Next, we examined the function of Mic60 in cancer. Using a proteomics screen in PRAD PC3 cells, we identified 119 high-confidence mitochondrial proteins that associate with Mic60 (Fig. 1*D*). Bioinformatics analysis of this dataset identified multiple regulators of mitochondrial membrane transport and organization, protein sorting, Ca^2+^ homeostasis, and oxidative phosphorylation ( *SI Appendix*, Fig. S1*E* ). Therefore, we sought to reproduce the phenotype of Mic60-low tumors (Fig. 1*A* and *SI Appendix*, Fig. S1 *A*–*C* ) by generating clones of PC3 or GBM LN229 cells with silencing of Mic60 by short hairpin RNA (shRNA) or CRISPR-Cas9 ( *SI Appendix*, Fig. S2*A* , *Top*). Small interfering RNA (siRNA) sequences targeting Mic60 were also characterized in PC3 cells, normal diploid fibroblasts, MRC5, breast adenocarcinoma MDA231, and osteosarcoma HT1080 cells ( *SI Appendix*, Fig. S2*A* , *Bottom*).

Using these approaches, silencing of Mic60 caused a catastrophic collapse of mitochondrial integrity in tumor cells, with disassembly of tubular network and cristae organization (Fig. 1*E*). This was accompanied by acute mitochondrial damage, characterized by increased outer membrane permeability (Fig. 1*F*) and depolarization of the inner membrane (Fig. 1*G* and *SI Appendix*, Fig. S2*B* ). As a result, Mic60-depleted tumor cells exhibited decreased oxygen consumption rates ( *SI Appendix*, Fig. S2*C* ), with lower basal and maximal respiration ( *SI Appendix*, Fig. S2*D* ), reduced adenosine triphosphate (ATP) production ( *SI Appendix*, Fig. S2 *E* and *F* ), and increased phosphorylation of AMPK, a marker of cellular starvation ( *SI Appendix*, Fig. S2*G* ). Despite a modest increase in antioxidant glutathione (GSH), these cells showed acute oxidative stress with a decreased GSH:glutathione disulfide (GSSG) ratio ( *SI Appendix*, Fig. S3*A* ), heightened production of total and mitochondrial reactive oxygen species (ROS) ( *SI Appendix*, Fig. S3*B* ), and increased expression of γH2AX ( *SI Appendix*, Fig. S3*C* ), as well as formation of subnuclear γH2AX foci ( *SI Appendix*, Fig. S3*D* ). Consistent with loss of mitochondrial integrity, Mic60-low tumor cells activated quality-control mechanisms of autophagy with punctate GFP-LC3 staining ( *SI Appendix*, Fig. S3*E* ), processing of LC3 to a lipidated form, and upregulation of p62, i.e., sequestosome ( *SI Appendix*, Fig. S3*F* ). Mitophagy was also induced in these settings, as judged by increased MitoKeima red fluorescence reporter activity ( *SI Appendix*, Fig. S3*G* ), loss of mitochondrial mass ( *SI Appendix*, Fig. S3*H* ), and degradation of mitochondrial outer membrane proteins ( *SI Appendix*, Fig. S3*I* ). Silencing of p62 was insufficient to restore outer membrane proteins or mitochondrial mass after Mic60 depletion.

### Requirement of Mic60 for Tumor Cell Proliferation.

**Fig. 2.**
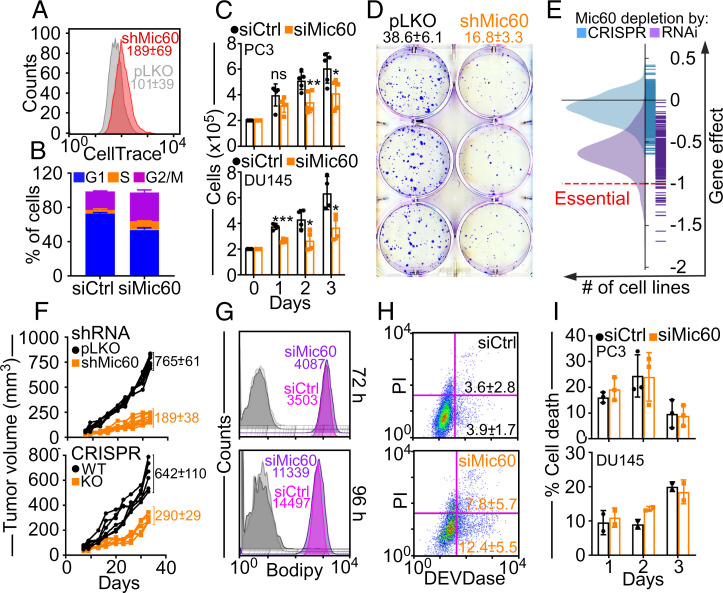
Requirement of Mic60 for tumor cell proliferation. (*A*) PC3 cells transduced with pLKO or shMic60 were labeled with CellTrace violet and analyzed by flow cytometry. The MFI of three independent experiments (mean ± SD) are shown. (*B*) PC3 cells transfected with siCtrl or siMic60 were analyzed by propidium iodide (PI) staining and flow cytometry after 7 d. The percentage of cells in the various cell cycle phases (mean ± SD) is shown (*n* = 4). (*C*) PC3 (*Top*) or PRAD DU145 (*Bottom*) cells as in *B* were analyzed for cell proliferation by direct cell counting at the indicated time intervals. Mean ± SD (*n* = 4 to 5). PC3, *P = 0.02; **P = 0.008. DU145, *P = 0.01 to 0.02; ***P = 0.0001. ns, not significant. (*D*) PC3 cells as in *A* were analyzed for colony formation (mean ± SD) by crystal violet staining and light microscopy. Representative images are shown (*n* = 6). *P* < 0.0001. (*E*) DepMap Project analysis of the effect of Mic60 depletion by RNAi or CRISPR on tumor cell proliferation. (*F*) PC3 cells as in *A* (*Top*) or wild-type (WT) or Mic60 knockout (KO) PC3 cells (*Bottom*) were injected subcutaneously in immunocompromised NSG mice and flank tumor growth was quantified at the indicated time intervals. Each line corresponds to an individual tumor. The mean ± SD of tumor growth (mm^3^) at d 35 is indicated (*n* = 6 to 8). *P* < 0.0001. (*G*) PC3 cells transfected with siCtrl or siMic60 were labeled for oxidized lipids (Bodipy) and analyzed after 72 h (*Top*) or 96 h (*Bottom*) by flow cytometry. MFIs of a representative experiment are indicated (*n* = 3). Gray shades, unstained cells. (*H*) PC3 cells as in *G* were analyzed for DEVDase activity/PI staining by multiparametric flow cytometry. The percentage of cells (mean ± SD) in early (*Bottom right*) or late (*Top right*) apoptosis are indicated (*n* = 3). (*I*) PC3 cells as in *G* were analyzed for cell death at the indicated time intervals by Trypan blue exclusion and direct cell counting. Mean ± SD (*n* = 2 to 3).

Based on these results, we next asked if the loss of mitochondrial fitness induced by Mic60 depletion affected tumor functions. Consistent with DNA damage ( *SI Appendix*, Fig. S3 *C* and *D* ), Mic60-depleted cells exhibited slower cell cycle progression (Fig. 2*A*) and accumulation of cells with G2/M DNA content throughout a 7-d culture (Fig. 2*B*). This resulted in a reduced proliferation of normal and tumor cell types (Fig. 2*C* and *SI Appendix*, Fig. S4*A* ), as well as an inhibition of colony formation (Fig. 2*D*). An analysis of the DepMap Portal (23) revealed dependency scores < 1 for all tumor types examined after Mic60 silencing by RNA interference (RNAi) or CRISPR-Cas9 (Fig. 2*E* and *SI Appendix*, Fig. S4*B* ), consistent with a general requirement of Mic60 for tumor cell proliferation. Accordingly, PC3 clones with reduced Mic60 levels by shRNA or CRISPR-Cas9 formed slow-growing superficial tumors in immunocompromised mice (Fig. 2*F*) with decreased *K*_i_-67 reactivity ( *SI Appendix*, Fig. S4*C* ), a marker of cell proliferation. Conversely, control PC3 cells formed exponentially growing flank tumors (Fig. 2*F*) with high *K*_i_-67 staining ( *SI Appendix*, Fig. S4*C* ).

Despite cellular damage and activation of mitophagy, tumor cells with reduced Mic60 did not upregulate ferroptosis-associated genes ( *SI Appendix*, Fig. S4*D* ), a type of cell death induced by mitochondrial stress, and oxidized lipid content, a marker of ferroptosis, was unchanged compared to control cultures (Fig. 2*G*). Similarly, Mic60 depletion only modestly affected mitochondrial apoptosis, as quantified by caspase (DEVDase) activity (Fig. 2*H*) or hypodiploid DNA content and flow cytometry ( *SI Appendix*, Fig. S4*E* ). Finally, no significant differences in necroptotic cell death were observed in control or Mic60-silenced cells over a 3-d (Fig. 2*I*) or 5-d culture ( *SI Appendix*, Fig. S4*F* ), by analysis of plasma membrane integrity and light microscopy.

### Mic60 Depletion Induces a Unique Innate Immunity and Cytokine/Chemokine Gene Signature.

**Fig. 3.**
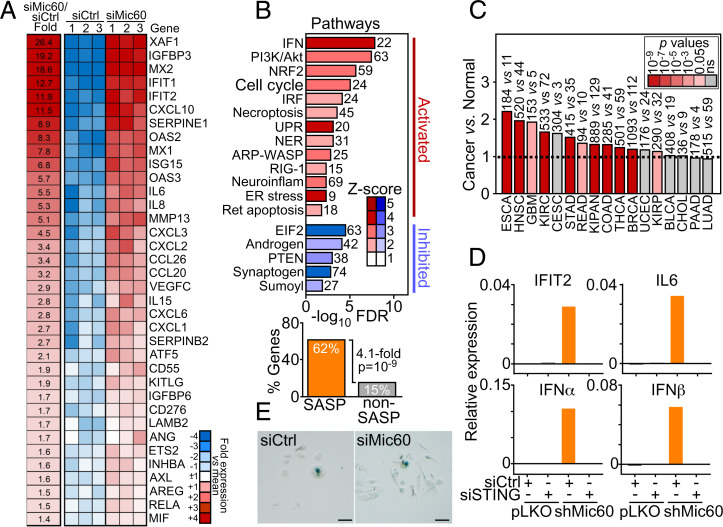
A Mic60 transcriptome in cancer. (*A*) PC3 cells transfected with siCtrl or siMic60 were analyzed by RNA-Seq, and relative fold changes in gene expression (false discovery rate [FDR] < 5%) were visualized in a heatmap. (*B*) Enrichment analysis of genes significantly different (FDR < 5%) between siCtrl and siMic60 from RNA-Seq results. Ingenuity pathway analysis (*Top*, canonical pathways) of pathways activated or inhibited after Mic60 knockdown. The number of genes affected and activation Z-scores are indicated. The Fisher exact test was used to test the significance of the enrichment of the SASP-like gene group, and the *P* value with fold enrichment of SASP-like vs. non-SASP gene products among the list of significant genes is shown. (*Bottom*) (*C*) The complete Mic60 transcriptome comprising 52 IFN/SASP-like genes was averaged across all TCGA tumors, and average levels were examined for differential expression in cancer vs. normal samples (ratio). The number of tumor and normal tissue samples is indicated per each condition. The broken line indicates ratio of 1, and the color scale indicates significance by *P* value. (*D*) PC3 cells transduced with pLKO or shMic60 were transfected with siCtrl or STING-directed siRNA (siSTING) and analyzed for the expression of representative genes in the Mic60 transcriptome by RT-PCR. Representative experiment is shown (*n* = 2). (*E*) PC3 cells as in *A* were analyzed for β-galactosidase staining and light microscopy (representative images). Scale bars, 100 μm.

Next, we looked at potential mechanisms of cellular adaptation in Mic60-low tumors. By RNA sequencing (RNA-Seq), Mic60 silencing induced unique transcriptional changes in PC3 cells with upregulation of a type I interferon (IFN) response and cytokines/chemokines reminiscent of a senescence-associated secretory phenotype (SASP) (Fig. 3*A*) (24). The Mic60 transcriptome activated in these settings also comprised PI3K/Akt signaling (see below) as well as pathways of genomic integrity (NER), endoplasmic reticulum stress, unfolded protein response (UPR), pattern recognition (RIG-1), and cytoskeletal (ARP-WASP) remodeling (Fig. 3*B*). In contrast, Mic60 depletion suppressed eIF2α signaling (Fig. 3*B* and see below).

In validation experiments, Mic60-depleted cells upregulated SASP-like cytokines (IL6, IL8, IL18, and IL1α), chemokines (CXCL2, CXCL3), protease (MMP13), and growth factor modulators (IGFBP7, IGFBP3) ( *SI Appendix*, Fig. S5*A* ), as well as effectors of IFN signaling, IFIT1, IFIT3, MX1, OAS1, ISG15, and IFITM1 ( *SI Appendix*, Fig. S5*B* ) by RT-PCR. IL23 and MMP1 were not affected ( *SI Appendix*, Fig. S5*A* ). Similar results were obtained at the protein level, as Mic60 silencing increased the expression of MX2 and MMP13 by Western blotting ( *SI Appendix*, Fig. S5*C* ) and heightened the release of cytokines (IL6, IL8, CXCL10), protease (MMP13), and IFNs (IFNα and IFNβ) in the cell supernatant compared to control transfectants ( *SI Appendix*, Fig. S5*D* ). Finally, TCGA analysis demonstrated that all 52 IFN/SASP-like genes of the Mic60 transcriptome were significantly upregulated in Mic60-low tumors of the head and neck, brain (GBM), colon, rectum, kidney, and thyroid compared to normal tissues (Fig. 3*C*).

Mechanistically, siRNA silencing of STING ( *SI Appendix*, Fig. S5*E* ), a key regulator of mitochondrion-directed innate immunity, abolished the increase in cytokine mRNA levels after Mic60 loss (Fig. 3*D*). In addition, Mic60 depletion was accompanied by an increased phosphorylation of STAT1 (Ser727) and extracellular release of HMGB1 ( *SI Appendix*, Fig. S5*F* ), which are two effectors of IFN signaling. In contrast, senescence-associated β-galactosidase staining was unchanged in control or Mic60-depleted cultures (Fig. 3*E*).

### Mic60 Regulation of Tumor Cell Invasion and Metastasis.

**Fig. 4.**
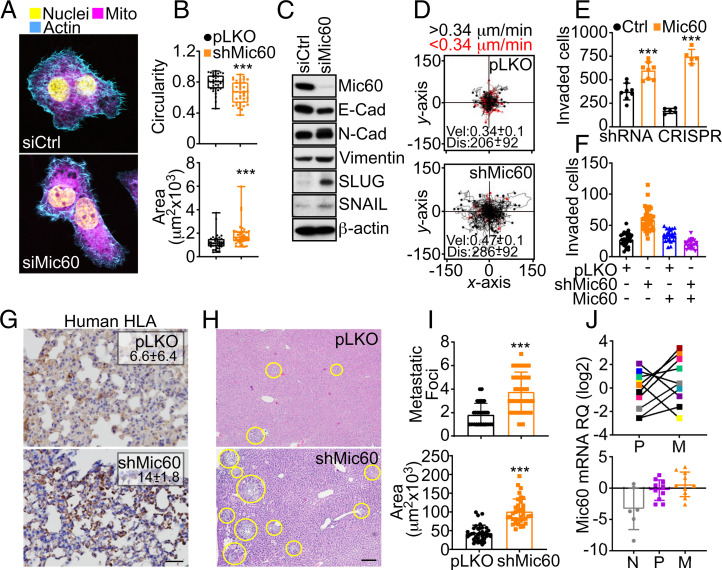
Mic60 regulation of tumor cell motility and metastasis. (*A*) PC3 cells transfected with siCtrl or siMic60 were analyzed by fluorescence microscopy. Representative images are shown. (*B*) PC3 cells transduced with pLKO or shMic60 were analyzed for circularity (*Top*; 1 = perfect sphere) and surface area (*Bottom*). Each symbol corresponds to an individual determination. Min to max values are indicated (pLKO, *n* = 36; shMic60, *n* = 39). ***P < 0.0001 to 0.0009. (*C*) PC3 cells as in *B* were analyzed by Western blotting. (*D*) PC3 cells transduced with pLKO or shMic60 were analyzed for single-cell motility in 2D contour plots by time-lapse video microscopy. Each tracing corresponds to the movements of an individual cell (pLKO, *n* = 90; shMic60, *n* = 94). The average speed of cell movements (velocity [Vel], μm/min) and distance traveled (Dis; μm) are indicated. Mean ± SD (*n* = 3). *P* < 0.0001. The cutoff velocities for slow-moving (red, <0.34 μm/min) or fast-moving (black, >34 μm/min) cells are indicated. (*E*) PC3 cells as in *B* or WT or Mic60 KO PC3 cells were analyzed for invasion across Matrigel-coated inserts and quantified. Mean ± SD (*n* = 4 to 5). ***P < 0.0001 to 0.0002. (*F*) PC3 cells as in *B* were transfected with vector or Mic60 cDNA and analyzed for Matrigel invasion. Two independent experiments were conducted (*n* = 21 to 43). (*G*) PC3 cells as in *B* were grown as superficial xenograft tumors in immunocompromised NSG mice (*n* = 6 to 8), and lungs from the indicated animal groups were harvested after 35 d and analyzed for human leukocyte antigen (HLA) staining by IHC. The % area of HLA reactivity (mean ± SD, *n* = 4 to 9) is indicated. *P* = 0.007. Scale bar, 100 μm. (*H* and *I*) PC3 cells as in *B* were injected into the spleen of NSG mice, and liver samples harvested after 11 d were stained with hematoxylin/eosin (*H*, representative images), and the number (*I*, *Top*) and surface area (*I*, *Bottom*) of metastatic foci were quantified by morphometry. Mean ± SD (*n* = 30 to 36). ***P < 0.0001. Yellow circles, liver metastases. Scale bar, 200 μm. (*J*) Matched patient samples of LUAD representative of normal parenchyma (N), primary tumor (P), or metastasis (M) were analyzed for differential Mic60 expression by RT-PCR. *Top*, individual sample analysis; *Bottom*, mean ± SD.

SASP signaling has been associated with increased tumor cell invasion and metastasis (25). Accordingly, Mic60 depletion changed the morphology of PC3 cells to a flattened, elongated, and spindle-shaped appearance, characterized by rearrangement of the actin cytoskeleton and redistribution of mitochondria to the cortical cytoskeleton (Fig. 4*A*). This was associated with reduced cellular roundness, increased surface area (Fig. 4*B*), and the appearance of epithelial-mesenchymal transition (EMT) markers, including E- and *N*-cadherin switch, and upregulation of vimentin, SLUG, and SNAIL (Fig. 4*C*). Functionally, Mic60 depletion enhanced FA turnover ( *SI Appendix*, Fig. S6*A* ), increasing the fraction of new and decayed FA, while reducing stable FA ( *SI Appendix*, Fig. S6*B* ). This resulted in a greater speed of single-cell movements, longer distance traveled by individual cells (Fig. 4*D*), and accelerated directional cell motility in a “wound” closure assay, shortening the half-time (t1/2) of wound closure from 16.3 h to 6.3 h ( *SI Appendix*, Fig. S6 *C* and *D* ). In addition, silencing of Mic60 increased tumor cell invasion across Matrigel (Fig. 4*E* and *SI Appendix*, Fig. S6*E* ), whereas re-expression of Mic60 complementary DNA (cDNA) in these settings normalized Matrigel invasion (Fig. 4*F*). As for signaling requirements, Mic60 silencing increased the phosphorylation of Focal Adhesion Kinase (FAK) ( *SI Appendix*, Fig. S6*F* ), and FAK targeting by siRNA ( *SI Appendix*, Fig. S6*G* ) or a small-molecule inhibitor (FA*K*_i_) normalized FA dynamics ( *SI Appendix*, Fig. S6*B* ) and restored the single-cell motility of PC3 ( *SI Appendix*, Fig. S6 *H* and *I* ) or LN229 ( *SI Appendix*, Fig. S6 *J* and *K* ) cells to levels of control transfectants.

Although impaired in primary tumor growth (Fig. 2*F*), superficial flank tumors of Mic60-depleted PC3 cells gave rise to increased metastatic dissemination to the lungs of immunocompromised mice (Fig. 4*G*). As a second, independent model of metastasis, we injected control or Mic60-depleted PC3 cells in the spleen of immunocompromised animals and looked at liver metastasis after 11 d. Here, Mic60-silenced PC3 cells generated more numerous and larger liver metastases compared to controls (Fig. 4 *H* and *I*). Based on these data, we next looked at matched patient samples of primary and metastatic LUAD. In this comparison, Mic60 mRNA levels increased in metastases compared to the primary tumor (Fig. 4*J*, *Top*) and the nonneoplastic lung tissue (Fig. 4*J*, *Bottom*), suggesting that Mic60 becomes re-expressed at established metastatic sites to support tumor cell proliferation.

### Regulation of Mitochondrial Dynamics and Cell Movements by Mic60.

The mechanism(s) underlying increased tumor cell motility after Mic60 targeting were next investigated. Despite the loss of mitochondrial fitness, Mic60 depletion stimulated mitochondrial dynamics in LN229 cells ( *SI Appendix*, Fig. S7 *A* and *B* ) and less consistently in other tumor types, resulting in higher rates of mitochondrial fission ( *SI Appendix*, Fig. S7*C* ). Mitochondrial fusion was less affected ( *SI Appendix*, Fig. S7 *B* and *C* ). As a result, Mic60-depleted cells exhibited heightened subcellular mitochondrial trafficking, with a longer distance traveled by individual mitochondria ( *SI Appendix*, Fig. S7*D* ) and increased accumulation of mitochondria at the cortical cytoskeleton of LN229 and PC3 cells compared to controls ( *SI Appendix*, Fig. S7 *E* and *F* ).

Mechanistically, siRNA silencing of mitochondrial GTPase RHOT1 or RHOT2 ( *SI Appendix*, Fig. S8*A* ), which mediate mitochondrial trafficking in tumors (26), normalized the speed of mitochondrial movements and the distance traveled by individual mitochondria after Mic60 depletion ( *SI Appendix*, Fig. S8*B* ). Buffering oxidative stress gave similar results, as the reconstitution of Mic60-silenced cells with antioxidant Prx3 (27) corrected the increase in single-cell motility ( *SI Appendix*, Fig. S8*C* ), lowered the speed of cell movements and the total distance traveled by individual cells to levels of control cultures ( *SI Appendix*, Fig. S8*D* ), and reversed the increase in Matrigel invasion in these settings ( *SI Appendix*, Fig. S8*E* ). Reconstitution of Mic60-silenced cells with a mitochondrial-targeted superoxide scavenger, MitoTempo, also normalized tumor cell invasion to control levels ( *SI Appendix*, Fig. S8*F* ).

### Adaptive GCN2-Akt Signaling as Therapeutic Vulnerability in Mic60-low Tumors.

**Fig. 5.**
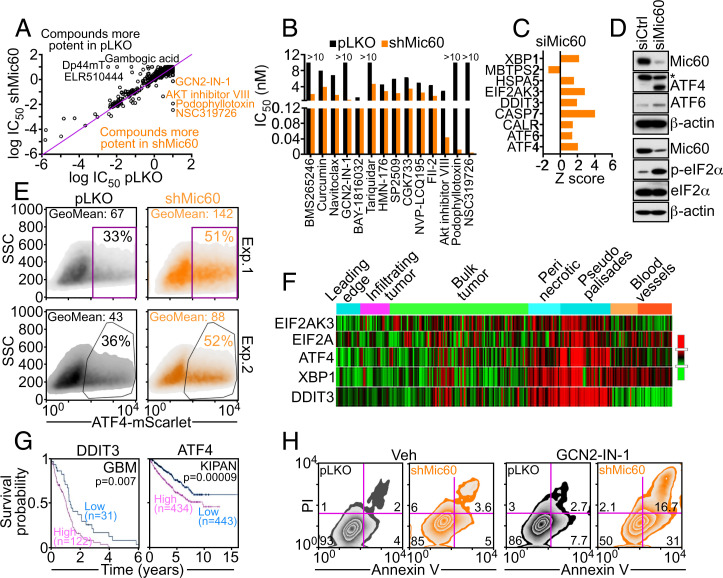
Therapeutic vulnerability of GCN2/Akt signaling exposed by Mic60 depletion. (*A* and *B*) PC3 cells transduced with pLKO or shMic60 were analyzed in a high-throughput drug screening (*A*), and candidate compounds with differential tumor cell killing in shCtrl- or shMic60-transduced cultures (*B*) were analyzed based on IC_50_ values (nM). (*C*) PC3 cells transfected with siMic60 were analyzed for differential ISR gene expression by RNA-Seq. A representative experiment is shown. (*D*) PC3 cells transfected with siCtrl or siMic60 were analyzed by Western blotting. p, phosphorylated. (*E*) PC3 cells as in *A* and *B* were transfected with ATF4-mScarlet nuclear reporter gene and analyzed by flow cytometry in two independent experiments (Exp). The percentage of cells in gated regions is indicated. (*F*) The indicated ISR regulators were analyzed for spatial distribution in patient-derived GBM samples (Ivy Glioblastoma Atlas Project) by RNA-Seq. The various intratumoral compartments are indicated. (*G*) Kaplan-Meier survival curves for differential expression of DDIT3 or ATF4 in Mic60-low GBM or pan-kidney cancer cohort comprising kidney chromophobe, kidney renal clear cell carcinoma, and kidney renal papillary cell carcinoma (KIPAN) in the Human Protein Atlas database. The number of patients per each condition and *P* values are indicated. (*H*) PC3 cells as in *A* and *B* were incubated with vehicle (Veh; *H*) or GCN2-IN-1 small-molecule inhibitor to GCN2 and analyzed for Annexin V/PI staining by multiparametric flow cytometry. The percentage of cells in each quadrant is indicated in a representative experiment (*n* = 2).

Finally, we asked if adaptive mechanisms activated in Mic60-low tumors exposed actionable therapeutic vulnerabilities. In a small-molecule drug screen, antagonists of Akt (Akt inhibitor VIII) or General Control Nonderepressible 2 kinase GCN2 (GCN2-IN-1) killed Mic60-silenced PC3 cells more efficiently than control cultures (Fig. 5 *A* and *B*). Natural compounds, podophyllotoxin and curcumin, p53 reactivator NSC319726, Bcl2 pathway inhibitor, Navitoclax, P glycoprotein antagonist, Tariquidar, multi-CDK inhibitor, NVP-LCQ195 and histone lysine-specific demethylase-1 inhibitor, and SP2509 also showed preferential killing against Mic60-low PC3 cells (Fig. 5*B*). Consistent with these data, Mic60 silencing increased Akt phosphorylation ( *SI Appendix*, Fig. S9*A* ), with downstream activation of Akt targets PDK1, BAD, p70S6K, and p27 quantified in a phosphoarray screen ( *SI Appendix*, Fig. S9 *B* and *C* ). Functionally, treatment with Akt inhibitor VIII or another small molecule Akt antagonist, MK2206, suppressed proliferation selectively of Mic60-depleted PC3 cells compared to control transfectants ( *SI Appendix*, Fig. S9*D* ).

GCN2 is a critical eIF2α kinase in the integrated stress response (ISR). Accordingly, ISR effectors ATF4, ATF6, eIF2AK3, and calreticulin were selectively upregulated after Mic60 depletion by RNA-Seq analysis (Fig. 5*C*). This was accompanied by an increased expression of ATF4 and ATF6 by Western blotting (Fig. 5*D*) and translocation of transcriptionally active ATF4 to the nucleus of Mic60-low PC3 cells compared to parental cultures (Fig. 5*E*). Consistent with these data, Mic60 silencing induced a strong phosphorylation of eIF2α in PC3 cells, whereas total eIF2α was unaffected (Fig. 5*D*). Bioinformatics analysis of the Ivy Glioblastoma Atlas Project (https://glioblastoma.alleninstitute.org) demonstrated that ISR regulators EIF2AK2, EIF2A, ATF4, XBP1, and DDIT3 were differentially increased in Mic60-low GBM and spatially localized within “pseudopalisades,” which are hypoxic hypercellular structures associated with greater invasiveness (Fig. 5*F*). Furthermore, high levels of ISR effectors DDIT3 and ATF4 correlated with shortened patient survival in Mic60-low GBM and kidney cancer (Fig. 5*G*) but not Mic60-high BRCA and LUAD ( *SI Appendix*, Fig. S9*E* ). Functionally, a small-molecule GCN2 inhibitor (GCN2-IN-1) inhibited proliferation (pLKO, 3.3 × 10^5^ ± 0.62 × 10^5^ cells; shMic60, 1.95 × 10^5^ ± 0.4 × 10^5^ cells; *P* = 0.009) and activated Annexin V-associated apoptosis (Fig. 5*H*) and caspase-dependent cell death (pLKO, 8.4 ± 6.3%; shMic60, 23.1 ± 5.6%; *P* = 0.04) preferentially in Mic60-silenced PC3 cells compared to control cultures. As an independent approach, siRNA silencing of GCN2 ( *SI Appendix*, Fig. S9*F* ) also increased cell death selectively in Mic60-silenced cultures ( *SI Appendix*, Fig. S9*G* ).

## Discussion

In this study, we have shown that Mic60, an essential scaffold of mitochondrial structure, is heterogeneously expressed and often reduced in human cancer compared to normal tissues. As modeled in tumor cell lines, even a partial reduction in Mic60 levels was sufficient to induce an acute loss of mitochondrial fitness, leading to bioenergetics defects, cellular starvation, and oxidative stress. Despite the activation of quality-control measures of autophagy and mitophagy, tumor cells harboring such extensively damaged, ghost mitochondria managed to evade cell death, slowed down cell proliferation, and activated mitochondrial dynamics to fuel increased cell invasion and metastasis. This response was accompanied by the expression of an IFN/SASP-like transcriptome, as well as adaptive activation of GCN2/Akt survival signaling, which provided an actionable therapeutic target in these metastasis-prone tumors.

The basis for the heterogeneous expression of Mic60 in cancer remains to be elucidated. This may result from mitochondrial and/or environmental stress conditions associated with tumor growth, including defective oxidative phosphorylation (28), or alternatively, mechanisms of tumor evolution, as suggested here by Mic60 downregulation during differentiation of patient-derived GBM neurospheres.

Irrespectively, decreased Mic60 levels generate subpar, ghost mitochondria that bear striking similarities to the mitochondrial defects seen in aging (29). Consistent with this parallel, in vivo models of aging have been associated with reduced Mic60 levels (30), heightened SASP signaling (31), mitochondria-to-nuclei retrograde gene expression (32), and a general, proinflammatory environment (33). However, key differences between aged and Mic60-low mitochondria were also noted (31). Aside from distinct profiles of cytokine induction, Mic60-depleted tumor cells were negative for senescence-associated β-galactosidase and did not undergo permanent G1 cell cycle arrest, and gene expression changes in these settings were unrelated to p53 status.

A unique adaptive response of Mic60-low tumors was the upregulation of a nuclear transcriptome combining a type I IFN response characteristic of innate immunity (34) and cytokines/chemokines reminiscent of SASP signaling (25). Mechanistically, mitochondrial stressors induced by Mic60 loss, such as ROS (35), loss of membrane integrity (36), and energy starvation (37), have all been associated with mitochondria-to-nuclei retrograde gene expression (38). Activation of a type I IFN response in these settings fits well with a key role of mitochondria in innate immunity (39), in line with the activation of STAT1 and the requirement of STING for cytokine production observed here. While there is evidence that SASP-associated cytokine/chemokine signaling promotes tumor growth, favors an immunosuppressive microenvironment, and enhances metastasis (25), the role of a type I IFN response in cancer is likely time- and context-specific. Whereas acute IFN signaling has been associated with antitumor immunity and improved treatment responses (34), sustained inflammatory conditions are protumorigenic (40) and chronic IFN stimulation enables myeloid-directed immunosuppression (41) and propagation of cancer stemness (42).

Consistent with this scenario, Mic60-low tumors switched from a proliferative to a highly motile and prometastatic phenotype, contributed by EMT, sustained FAK phosphorylation, and heightened mitochondrial dynamics (8). Described as phenotype switching (43), this reversible transition between proliferative and migratory states has been proposed as a potential escape mechanism for tumor cells to leave a stress-laden, unfavorable microenvironment and colonize distant sites, i.e., metastasis (44). Regulators of mitochondrial dynamics, such as syntaphilin (27), FUNDC1 (45), and now Mic60 depletion (this study), are important mediators of phenotype switching, reprogramming oxidative bioenergetics, and redox balance to promote heightened cell migration and invasion at the expense of cell proliferation. Consistent with this model, oxidative stress generated in Mic60-low tumors was a key mediator of increased mitochondrial trafficking and tumor cell movements, in keeping with a central role of ROS in tumor cell motility (46), EMT, and metastasis (47).

Despite an extensive loss of mitochondrial fitness, activation of autophagy/mitophagy, and high ROS production, Mic60-low tumor cells managed to persist likely through the activation of compensatory cell survival mechanisms. The induction of GCN2/ISR as well as Akt signaling (48, 49) observed in these settings appears ideally poised to adjust metabolism under stress (50, 51), preserve mitochondrial integrity (22, 52) and oppose cell death (53, 54). Although correlating with shortened patient survival, a potential dependence of Mic60-low tumors to GCN2/ISR/Akt adaptive signaling was therapeutically exploitable, and proof-of-concept studies shown here demonstrated that pharmacologic or genetic targeting of this pathway can restore mitochondrial cell death and inhibit proliferation selectively of Mic60-low tumor cells.

In summary, we have shown that persistent, acutely degraded ghost mitochondria are major signaling hubs in cancer, driving multiple, adaptive responses of nuclear gene expression, ISR activation, and suppression of mitochondrial cell death to enable metastatic competence. This reinforces the role of mitochondrial reprogramming as an important therapeutic target in cancer (55), especially in hard-to-treat and metastasis-prone malignancies with currently limited therapeutic options.

## Materials and Methods

### Patient Samples.

Primary patient samples arranged in a universal TMA were examined for differential Mic60 expression by IHC, as described (56). Archival tissues and clinical records were obtained from Fondazione Istituto di Ricovero e Cura a Carattere Scientifico (IRCCS) Ca’ Granda Hospital in Milan, Italy, under a protocol approved by the Institutional Review Boards (IRBs) of Fondazione IRCCS Ca’ Granda-Ospedale Maggiore Policlinico (code 179/2013). Because of the retrospective nature of this study and the use of data anonymization practices, the need for written informed consent was waived. For the glioma series, fresh-frozen material was available from Fondazione IRCCS Ca’ Granda Hospital under IRB protocol 275/2013, and written informed consent from all patients was obtained before surgery. Clinically annotated patient samples with a confirmed histologic diagnosis of PDAC (*n* = 5) were obtained from the archival database of the Department of Pathology at Yale New Haven Hospital upon approval from the Yale University IRB and examined for intratumoral heterogeneity of Mic60 expression by IHC.

### Proteomics.

Immune complexes of Mic60 or nonbinding immunoglobulin G (IgG) were precipitated from PC3 cells and separated by sodium dodecyl sulphate gel electrophoresis for ∼5 mm followed by fixing and staining with colloidal Coomassie. The region of the gel-containing proteins was excised and digested with trypsin. Tryptic peptides were analyzed by liquid chromatography-tandem mass spectrometry (LC-MS/MS) on a Q Exactive high-field (HF) mass spectrometer (Thermo Scientific) coupled with a Nano-ACQUITY ultra performance liquid chromatography (UPLC) system (Waters). Samples were injected onto a UPLC Symmetry trap column (180-μm inner diameter [i.d.] × 2 cm packed with 5-μm C18 resin; Waters), and tryptic peptides were separated by RP-HPLC on a BEH C18 nanocapillary analytical column (75-μm i.d. × 25 cm, 1.7-μm particle size; Waters) using a 90-min gradient. Eluted peptides were analyzed in data-dependent mode where the mass spectrometer obtained full MS scans from 400 to 2,000 *m/z* at 60,000 resolution. Full scans were followed by MS/MS scans at 15,000 resolution on the 20 most abundant ions. Peptide match was set as preferred, and the exclude isotopes option and charge-state screening were enabled to reject singly and unassigned charged ions. MS/MS spectra were searched using MaxQuant 1.6.5.0 (57) against the UniProt human protein database (October 2017). MS/MS spectra were searched using full tryptic specificity with up to two missed cleavages, static carbamidomethylation of Cys, variable oxidation of Met, and variable protein N-terminal acetylation. Consensus identification lists were generated with false discovery rates of 1% at protein and peptide levels. Undetected protein intensity values of 0 were floored to the value of 10^6^ (minimum nonzero detected intensity was 1,233,300), and a total of 1,534 detected proteins were taken for further annotation analysis. Proteins were then annotated as mitochondrial related using the MitoCarta 2.0 database, and 119 mitochondrial proteins detected with at least 5 peptides at an intensity over 10-fold versus IgG control were considered as Mic60-associated proteins.

### Mitochondrial Time-Lapse Videomicroscopy.

Cells (2 × 10^4^) growing on high optical quality glass-bottom 35-mm plates (MatTek Corporation) were incubated with 100 nM Mitotracker Deep Red FM dye for 1 h and imaged on a Leica TCS SP8 × inverted laser scanning confocal microscope using a 63× 1.40NA oil objective as described (58).

### Single-Cell Motility.

Cells (2 × 10^4^) were seeded in 4-well Ph+ Chambers (Ibidi) in complete growth medium and allowed to attach for 16 h at 37 °C. Time-lapse videomicroscopy was performed over 10 h, with a time-lapse interval of 10 min. Stacks were imported into Image J Fiji software for analysis, and at least 10 to 20 cells per condition were tracked using the Manual Tracking plugin for Image J Fiji. Tracking data were exported into the Chemotaxis and Migration Tool v. 2.0 (Ibidi) for graphing and calculation of the mean and SD of speed and accumulated distance of movement. For directional cell migration, wounds were made in monolayers of PC3 cells using a 10-μL pipette tip. Cell debris were washed off, and cultures were maintained in complete growth medium containing 10% fetal bovine serum at 37 °C and 5% CO_2_. Time-lapse imaging of migrating cells was performed using a TE300 inverted microscope (Nikon) equipped with an incubator set at 37 °C, 5% CO_2_, and 95% relative humidity. Each image was acquired using a 10× objective of the same fields at each 10-min interval for a total of 24 h.

### Small-Molecule Drug Screening.

Cell viability screening against the MedChem Express anti-cancer library (1,820 compounds) was performed using CellTiterGlo (Promega). PC3 cells stably transduced with pLKO or Mic60-directed shRNA (shMic60) were maintained in complete media, trypsinized, and plated (500 cells/well) in 40 μL of complete medium the day before the experiment in white, clear-bottom 384-well plates. A total of 50 nL of test compound was added to each well using the Janus MDT Nanohead (Perkin-Elmer). Each compound was screened at a final concentration of 10, 1, 0.1, and 0.01 μM. After a 72-h incubation at 37 °C in the presence of 5% CO_2_, 20 μL of the CellTiterGlo reagent was added to each well. After 15 min, luminescence was measured using the Envision Multimode plate reader (PerkinElmer). The raw data were normalized to % inhibition, where 0% is the relative light units (RLU) in the presence of dimethylsulfoxide, and 100% is the RLU in the presence of 1 μM bortezomib. Estimated half maximal inhibitory concentration (IC_50_) values for each compound were determined using nonlinear regression fits on the data to a one-site binding model in XlFit (ID Business Solutions Ltd. [IDBS]). Because only 4 data points were used in this calculation, the top and bottom of the curve was fixed to 100% and 0%, respectively, with a constant slope value of 1.

### Statistical Analysis.

Data are expressed as mean ± SEM or mean ± SD of multiple independent experiments or replicates of representative experiments out of a minimum of two or three independent determinations. Two-tailed Student’s *t* test or Wilcoxon rank sum test was used for two-group comparative analyses. For multiple-group comparisons, ANOVA or Kruskal–Wallis test with post hoc Bonferroni’s procedure were applied. All statistical analyses and graphing were performed using GraphPad software package (Prism 9.0) for Windows. A *P* value of <0.05 was considered statistically significant.

## Supplementary Material

Supplementary File

## Data Availability

All study data are included in the article and/or *SI Appendix* .
