# Supplementary material for: Ghost mitochondria drive metastasis through adaptive GCN2/Akt therapeutic vulnerability
Source: Proc Natl Acad Sci U S A. 2022 Feb 17;119(8):e2115624119. doi: 10.1073/pnas.2115624119 (PMC8872753; doi:10.1073/pnas.2115624119)
Supplement: Supplementary File [file pnas.2115624119.sapp.pdf]

GHOST MITOCHONDRIA DRIVE METASTASIS THROUGH ADAPTIVE GCN2/Akt THERAPEUTIC VULNERABILITY

Jagadish C. Ghosh, Michela Perego, Ekta Agarwal, Irene Bertolini, Yuan Wang, Aaron R. Goldman, Hsin-Yao Tang, Andrew V. Kossenkov, Catherine J. Landis, Lucia R. Languino, Edward F. Plow, Annamaria Morotti, Luisa Ottobrini, Marco Locatelli, David W. Speicher, M. Cecilia Caino, Joel Cassel, Joseph M. Salvino, Marie E. Robert, Valentina Vaira, and Dario C. Altieri

SUPPLEMENTARY INFORMATION

MATERIALS AND METHODS

Cells and cell cultures. Prostate adenocarcinoma PC3 and DU145, breast adenocarcinoma MDA231, human osteosarcoma HT1080, and glioblastoma LN229 cells were obtained from the American Type Culture Collection (ATCC, Manassas, VA) and maintained in culture according to the supplier's specifications. Normal human diploid MRC5 fibroblasts were also obtained from ATCC. Conditioned media used for cell migration and invasion experiments was prepared from exponentially growing NIH3T3 fibroblasts (ATCC) in DMEM supplemented with 4.5 g/l D-glucose, sodium pyruvate, 10 mM HEPES and 10% FBS for 48 h. For all cell types, cell passaging was limited to <40 passages from receipt. Cell lines were authenticated by STR profiling with AmpFlSTR Identifiler PCR Amplification Kit (Life Technologies) at the Genomics Shared Resource of The Wistar Institute. Mycoplasma free-cultures were confirmed at the beginning of the studies, and every 2 months afterwards by PCR amplification of cultures using Bioo Scientific Mycoplasma Primer Sets (cat#375501) and Hot Start polymerase (QIAGEN). Tumor types were transfected with the various siRNA at 25 nM in Lipofectamine RNAiMAX (Invitrogen) at a 1:1 ratio (vol siRNA 20 μM/vol Lipofectamine RNAiMAX). After 48-72 h, transfected cells were validated for target protein knockdown by Western blotting and processed for functional experiments. Clones of PC3 cells were generated by transfection of Mic60 guide RNA (5′-AAAACTCTTACCGATTTCTT-3′) using lentiCRISPR v2 (Addgene, cat. no. 52961) in the presence of lipofectamine 3000 followed by puromycin selection (2 μg/ml). PC3 cells transduced with empty vector were used as control and Mic60 expression was examined by Western blotting. Ex-vivo differentiation of patient derived GBM neurospheres was carried out as described (1).

Antibodies and reagents. Antibodies to VDAC, AMPKα, Thr172-phosphorylated AMPKα, FAK, Tyr925-phosphorylated FAK, STAT1, Tyr701-phosphorylated STAT1, IL6, XAF1, MX2, MMP13, STING, and HMGB1 were from Cell Signaling. Antibodies to Mic60, p62, RHOT1, and RHOT2 were purchased from Santa Cruz. Antibodies to Hsp60 (BD Biosciences), Prx3 (Invitrogen), TOM20 (Protein Tech), γH2AX (Millipore), Ki-67 (Ventana Medical Systems, Roche Group), and β-actin (Sigma-Aldrich) were also used. MitoTracker Green, Phalloidin Alexa488, Mitotracker-Deep Red FM and secondary antibodies used in immunofluorescence experiments were from Molecular Probes. MitoSOX Red (Life Technologies) or total CellRox Deep Red (Thermo Fisher) were used for ROS quantification. A glutathione assay kit was purchased from Cayman Chemical. A small molecule GCN2 inhibitor (GCN2-IN-1) was from MedChemExpress. Small molecule Akt inhibitors, MK2206 and Akt inhibitor VIII as well as MitoTempo were from Sigma. A pLVX-ATF4 mScarlet NLS lentivirus was from Addgene. Bodipy and CellTrace Violet fluorescence dyes were purchased from Thermo Fisher Scientific. The following siRNA sequences were used: control, ONTARGET plus nontargeting siRNA pool (Dharmacon D-001810), Mic60 (GUCUAGAAAUGAGCAGGUUUA). A STING-directed siRNA (siSTING) was from Sigma Aldrich (SASI_Hs02_00371843). The following human 3' UT (position 2459) Mic60 (IMMT)-directed shRNA TRCN0000135616 (shRNA sequence, TAAACCTGCTCATTTCTAGAC; target sequence, GTCTAGAAATGAGCAGGTTTA) was used to generate PC3 cells with stable Mic60 knockdown. An empty pLKO-based lentivirus was used as control, and selection of stable clones was carried out in the presence of puromycin (2 μg/ml).

Protein analysis. Protein lysates were prepared in 150 mM NaCl, 1% Triton X-100, 0.5% sodium deoxycholate, 0.1% SDS, 50 mM Tris, pH 8.0 in the presence of EDTA-free Protease

Inhibitor Cocktail (Roche) and Phosphatase Inhibitor Cocktail (Roche). Equal amounts of protein lysates were separated by SDS gel electrophoresis, transferred to PVDF membranes and incubated with primary antibodies of various specificities. Protein bands were visualized by chemiluminescence.

mRNA expression. RNA was extracted from the various cell types using Quick-RNA Microprep (Zymo Research) according to the manufacturer's instructions. cDNA was prepared with High32 Capacity cDNA Reverse Transcription Kit with RNase Inhibitor (ThermoFisher Scientific) and the reverse-transcription reaction performed on a BioRad T100 Thermal Cycler. Quantitative PCR was performed with SYBR™ Select Master Mix (ThermoFisher) on ABI Quant Studio 5 machine (ThermoFisher). The following primer sequences (FW, forward; RV, reverse) were used for amplification of the indicated target mRNAs: GATAGCCAGCCTAGAGGTATGG (**hu-IL18-FW**), CCTTGATGTTATCAGGAGGATTCA (**hu-IL18-RV**), GAGCCTTCTCTGCTCCCTGATA (**hu-IL23-FW**); GACTGAGGCTTGGAATCTGCTG (**hu-IL23-RV**); CGCTACAAAGTTGACTACGAGTC (**hu-IGFBP3-FW**); GTCTTCCATTTCTCTACGGCAGG (**hu-IGFBP3-RV**); CCTTGATGCCATTACCAGTCTCC (**hu-MMP13-FW**); AAACAGCTCCGCATCAACCTGC (**hu-MMP13-RV**); GGCAGAAAGCTTGTCTCAACCC (**hu-CXCL2-FW**); CTCCTTCAGGAACAGCCACCAA (**hu-CXCL2-RV**); TTCACCTCAAGAACATCCAAAGTG (**hu-CXCL3-FW**); TTCTTCCCATTCTTGAGTGTGGC (**hu-CXCL3-RV**); CCTGGAATGCTTACGGCAAGCT (**hu-IFIT3-FW**); GAGCATCTGAGAGTCTGCCCAA (**hu-IFIT3-RV**); GCCTTGCTGAAGTGTGGAGGAA (**hu-IFIT1-FW**); ATCCAGGCGATAGGCAGAGATC (**hu-IFIT1-RV**); GGCTTCATAGCATTCGCCTACTC (**hu-IFITM1-FW**);

AGATGTTCAGGCACTTGGCGGT (**hu-IFITM1-RV**); AGGAAAGGTGCTTCCGAGGTAG (**hu-OAS1-FW**); GGACTGAGGAAGACAACCAGGT (**hu-OAS1-RV**); CTCTGAGCATCCTGGTGAGGAA (**hu-ISG15-FW**); AAGGTCAGCCAGAACAGGTCGT (**hu-ISG15-RV**); GGCTGTTTACCAGACTCCGACA (**hu-MX1-FW**); CACAAAGCCTGGCAGCTCTCTA (**hu-MX1-RV**); GACTGTGCACTTGCTGGTGGAT (**hu-IL-6-FW**); ACTTCCTCACCAAGAGCACAGC (**hu-IL-6-RV**); GAGAGTGATTGAGAGTGGACCAC (**hu-IL-8-FW**); CACAACCCTCTGCACCCAGTTT (**hu-IL-8-RV**); AGAATCACCAGCAGCAAGTGTCC (**hu-MCP-1-FW**); TCCTGAACCCACTTCTGCTTGG (**hu-MCP-1-RV**); ATGAAGCAGCCCAGATGTGGAG (**hu-MMP-1-FW**); TGGTCCACATCTGCTCTTGGCA (**hu-MMP-1-RV**); CACTCACAGACCTGACTCGGTT (**hu-MMP-3-FW**); AAGCAGGATCACAGTTGGCTGG (**hu-MMP-3-RV**); CTCTTCTGCCTGCTGCACTTTG (**hu-TNFα-FW**); ATGGGCTACAGGCTTGTCACTC (**hu-TNFα 1-RV**); TGTATGTGACTGCCCAAGATGAAG (**hu-IL-1α-FW**); AGAGGAGGTTGGTCTCACTACC (**hu-IL-1α -RV**); CCACAGACCTTCCAGGAGAATG (**hu-IL-1β-FW**); GTGCAGTTCAGTGATCGTACAGG (**hu-IL-1β-RV**); GCCATCACCCAGGTCAGCAAG (**hu-IGFBP7-FW**); GGATTCCGATGACCTCACAGCT (**hu-IGFBP7-RV**).

RNA-Seq analysis. RNA-seq data was aligned using the bowtie2 (2) algorithm against hg19 human genome version and RSEM v1.2.12 software (3) was used to estimate read counts and RPKM values using gene information from Ensemble transcriptome version GRCh37.p13. Raw counts were used to estimate significance of differential expression difference between two experimental groups using DESeq2 (4). Overall gene expression changes were considered significant if passed FDR <5% thresholds. Gene set enrichment analysis was done using

QIAGEN's Ingenuity® Pathway Analysis software (IPA®, QIAGEN Redwood City, www.qiagen.com/ingenuity) using the "Canonical pathways" option. Pathways that passed significance of FDR <5% threshold and had significantly predicted activation state (|Z-score|>2) were reported. Enrichment of SASP genes in the list of significant genes was tested using Fisher Exact Test. For analysis of cancer cell dependency, we searched the Cancer Dependency Map website from the Broad Institute. CERES or DEMETER2 gene effect scores from DepMap public release 19Q3 were downloaded from https://depmap.org/portal/download/ (Oct 10 2019).

TCGA analysis. The TCGA tumor expression data was downloaded and log2-transformed. Averaged expression values for the Mic60 transcriptome (52 IFN/SASP-like genes) were tested in 28 tumor sets that had matching normal tissue RNA-seq data for differential expression between tumor and normal groups using unpaired t test. Results with $p<0.05$ were considered significant.

Transmission Electron Microscopy. PC3 cells were fixed with 2.5% glutaraldehyde, 2% paraformaldehyde in 0.1 M sodium cacodylate buffer for 16 h. After subsequent washed in the same buffer, samples were post-fixed in 2% osmium tetroxide for 1 h at 22°C and rinsed in water prior to en bloc staining with 2% uranyl acetate. After dehydration through a graded ethanol series, samples were infiltrated and embedded in Embed-812 (Electron Microscopy Sciences, Fort Washington, PA). Sections were stained with uranyl acetate and lead citrate and imaged on a JOEL 1010 electron microscope at 50,000x magnification.

Immunofluorescence. Cells were fixed in formalin/PBS (4% final concentration), pH 7.2, for 15 min at 22°C, permeabilized in 0.1% Triton X-100/PBS for 5 min, washed, and incubated in 5% normal goat serum (NGS, Vector Labs) diluted in 0.3 M glycine/PBS for 60 min. Primary antibodies against β-tubulin (diluted 1:200), mitochondrial cytochrome c oxidase subunit II MTCO2 (diluted 1:500) or TOM20 (1:100) were added in 5% NGS/0.3 M glycine/PBS and

incubated for 18 h at 4°C. After 3 washes in PBS, secondary antibodies conjugated to TRITC were diluted 1:500 in 5% NGS/0.3 M glycine/PBS and incubated with the various samples for 1 h at 22°C. Where indicated, F-actin was stained with phalloidin Alexa488 (1:200 dilution) for 30 min at 22°C. Slides were washed and mounted in DAPI-containing Prolong Gold mounting medium (Invitrogen). To determine the positioning of mitochondria at the cortical cytoskeleton, 45-48 cells were selected per each sample. For quantification of cortical mitochondria, mitochondria/F-actin composite images were analyzed in ImageJ. The F-actin channel was used to manually label the cell boundary and a belt extending from the boundary towards the inside of the cell was marked as "cortical mask". This cortical mask was subsequently applied to the mitochondrial channel to measure intensity at the cortical region and normalized to total mitochondrial intensity per cell and cell area.

FA dynamics. PC3 cells under the various conditions tested were plated on high optical quality 35-mm glass bottom plates and transduced with Talin-RFP BacMam virus for 18 h. Time-lapse videomicroscopy was carried out using a Leica TCS SP8 Scanning Laser Confocal Microscope system with an HCX PL APO CS 63× 1.40 NA oil UV objective. Acquisition of live cells using an integrated Leica LAS software was performed every 3 min per frame for a total interval of 2 h. Sequences were imported in ImageJ for further analysis. The initial and final frames were duplicated and assembled as composite images. FA were manually counted and classified (according to the presence in some or all the time frames) into three groups: decaying, newly formed and stable mature (merged areas). The analysis was carried out in 7 cells (about 150 FA complexes) per condition in 2 independent time-lapse experiments using a LASX software package.

Flow cytometry. For cell cycle analysis, PC3 cells under the various conditions tested were fixed in 70% cold ethanol, mixed by vortexing and maintained at 4°C for 2 h prior further processing. Before staining, cells were washed twice in PBS, pH 7.2, and mixed with 500 μl FxCycle PI/ribonuclease (RNase) staining solution/sample (Thermo Fisher) for 20 min at 22°C in the dark. After washing in PBS, samples were analyzed using a BD™ LSR II cytometer (BD). For proliferative rate, PC3 cells were stained with CellTrace™ Violet according to the manufacturer's instructions, seeded at $5x10^4$ cells/ml in RPMI containing 10% FBS and maintained in a humidified incubator with 5% CO_2 at 37°C After a maximum incubation of 7 d, cells were harvested and analyzed on a BD FACSCelesta™. Data were analyzed using FlowJo™ v10.7 after gating on live single cells. For apoptosis, cells were seeded at $1.25x10^5$/ml in a 6-well plate in complete medium for 16 h at 37°C. Cultures were incubated with or without GCN2-IN-1 (10 μM) or equivalent amount of DMSO (vehicle) for additional 24 h at 37°C. Cells were harvested by trypsinization and stained with FITC Annexin V Apoptosis Detection Kit I (BD Pharmingen™) according to the manufacturer's instruction. In some experiments, caspase-dependent apoptosis was quantified using DEVDase activity (CaspaTag Caspase 3,7 In Situ Assay Kit, Millipore Sigma, cat. #APT423) and multiparametric flow cytometry. Samples were acquired on a BD FACSCelesta™ Cell Analyzer and analyzed by FlowJo™ v10.7.

ROS. PC3 cells (4×10^5) under the various conditions tested were stained with mitochondrial superoxide indicator MitoSOX Red (5 μM) or total CellRox Deep Red (5 μM) for 10 min in complete medium, followed by washes in PBS, pH 7.2, and analyzed on a FACSCalibur flow cytometer. Intact cells were gated in the FSC/SSC plot to exclude small debris.

Cellular respiration. Oxygen Consumption Rates (OCR) were quantified using an Agilent Seahorse XFe96 analyzer (Agilent Technologies, Wilmington, DE). Briefly, PC3 cells ($3x10^4$) in

complete growth medium were plated in each well of a Seahorse XFe96 cell culture plate (80 μl volume) for 24 h at 37°C in 5% CO_2. The media was exchanged with XF base media (Agilent) supplemented with 2 mM glutamine, 1 mM sodium pyruvate and 10 mM glucose, pH 7.4, and equilibrated for 1 h at 37°C in a non-CO_2 incubator before the experiment. Metabolic rates were monitored under basal conditions (before any addition) and after addition of oligomycin (1 μM), FCCP (1 μM) and antimycin (0.5 μM), all dissolved in XF base media. The three drugs were injected into the XFe96 sequentially, and OCR were measured using three cycles of mixing (150 sec), waiting (120 sec) and measuring (210 sec). This cycle was repeated after each injection.

Mitochondrial membrane potential. PC3 cells were washed three times in PBS, pH 7.4, and analyzed on a FACSCalibur flow cytometer with the TMRE signal as FL1. Intact cells were gated in the FSC/SSC plot to exclude small debris. The resulting FL1 data were plotted on a histogram.

Mitophagy assay. Experiments were carried out using a FACS-based analysis of mitochondrial targeted Keima-Red fluorescence reporter (Addgene, cat. #56018). Briefly, PC3 cells stably expressing mitochondrial Keima-Red were transfected with control non-targeting siRNA or Mic60-directed siRNA, in the presence or absence of the mitochondrial uncoupler, FCCP. Cells were detached with trypsin, washed and suspended in PBS followed by analysis at 405 and 561 nm lasers and 610/20 filters on an LSR 18 flow cytometer. Intact cells were gated in the FSC/SSC plot to exclude small debris. A ratiometric value between phycoerythrin (PE)-Texas-Red (TR) and BV605 fluorescence intensity was derived for data quantification.

Tumor cell invasion. Experiments were carried out essentially as described (5) using Growth Factor Reduced Matrigel-coated 8 μm PET Transwell chambers (Corning). The various tumor

cell types were seeded in duplicate onto the coated Transwell filters ($1x10^5$ cells/well) in medium containing 0.1% BSA and conditioned media from NIH3T3 fibroblasts was placed in the lower chamber as chemoattractant. Cells were allowed to invade for 16 h, non-invading cells were scraped off the topside of the membranes, and invasive cells on the Transwell insert were fixed in methanol. Membranes were mounted in medium containing DAPI (Vector Labs) and analyzed by fluorescence microscopy. Five random fields at 10X magnification were collected for each Transwell membrane. Digital images were batch imported into ImageJ Fiji, thresholded and analyzed with the Analyze particle function.

Animal studies. Experiments were carried out in accordance with the recommendations in the Guide for the Care and Use of Laboratory Animals of the National Institutes of Health (NIH). Protocols were approved by the Institutional Animal Care and Use Committee (IACUC) of The Wistar Institute. All animals were included in the analysis. A liver metastasis model was performed essentially as described (5). Surgical procedures were carried out in isoflurane-anesthetized animals following aseptic techniques inside a biosafety cabinet, and a slow-release buprenorphine formulation was administered for pain relief. For these experiments, PC3 cells stably transduced with pLKO or shMic60 at 80% confluency were suspended in PBS, pH 7.2, and $1x10^6$ cells were injected (50 μl) in the spleen of anesthetized 6-8 weeks old male NOD SCID γ (NSG, NOD.Cg-Prkdcscid Il2rgtm1Wjl/SzJ) mice (Jackson Laboratory). After 11 d, animals were sacrificed and the number and surface areas of metastatic foci to the liver were quantified by morphometry. In other experiments, PC3 stably transduced with pLKO or shMic60 or, alternatively, wild type (WT) or Mic60 KO cells were engrafted s.c. on the flanks of NOD SCID γ mice (10 animals/group) and tumor growth was measured with a caliper throughout a 33-d interval. Tumors were excised at the end of the experiment and the percentage of Ki-67-

positive cells was quantified by IHC. Lung and liver samples collected from the various animal groups were analyzed for metastatic dissemination by human HLA reactivity and an IHC score was determined per each condition.

IHC. Four μm-thick sections from tissue blocks of human or mouse tissue samples were stained with primary antibodies to Mic60 (patient samples), HLA-1 or Ki-67 (mouse models) using using Benchmark Ultra Roche Ventana Immunostainer (Roche Group, Tucson, AZ) and diaminobenzidine (DAB) as a chromogen. All slides were counterstained with hematoxylin. Patient immunoreactivity in cases of lung, breast and colorectal adenocarcinoma was calculated as the percentage of positive cells out of the total number of cells examined from digital imaging reconstruction of stained tissue areas.

SUPPLEMENTARY REFERENCES

1. A. Di Cristofori *et al.*, The vacuolar H+ ATPase is a novel therapeutic target for glioblastoma. *Oncotarget* **6**, 17514-17531 (2015).
2. B. Langmead, S. L. Salzberg, Fast gapped-read alignment with Bowtie 2. *Nat Methods* **9**, 357-359 (2012).
3. B. Li, C. N. Dewey, RSEM: accurate transcript quantification from RNA-Seq data with or without a reference genome. *BMC Bioinformatics* **12**, 323 (2011).
4. M. I. Love, W. Huber, S. Anders, Moderated estimation of fold change and dispersion for RNA-seq data with DESeq2. *Genome Biol* **15**, 550 (2014).
5. M. C. Caino *et al.*, A neuronal network of mitochondrial dynamics regulates metastasis. *Nat Commun* **7**, 13730 (2016).

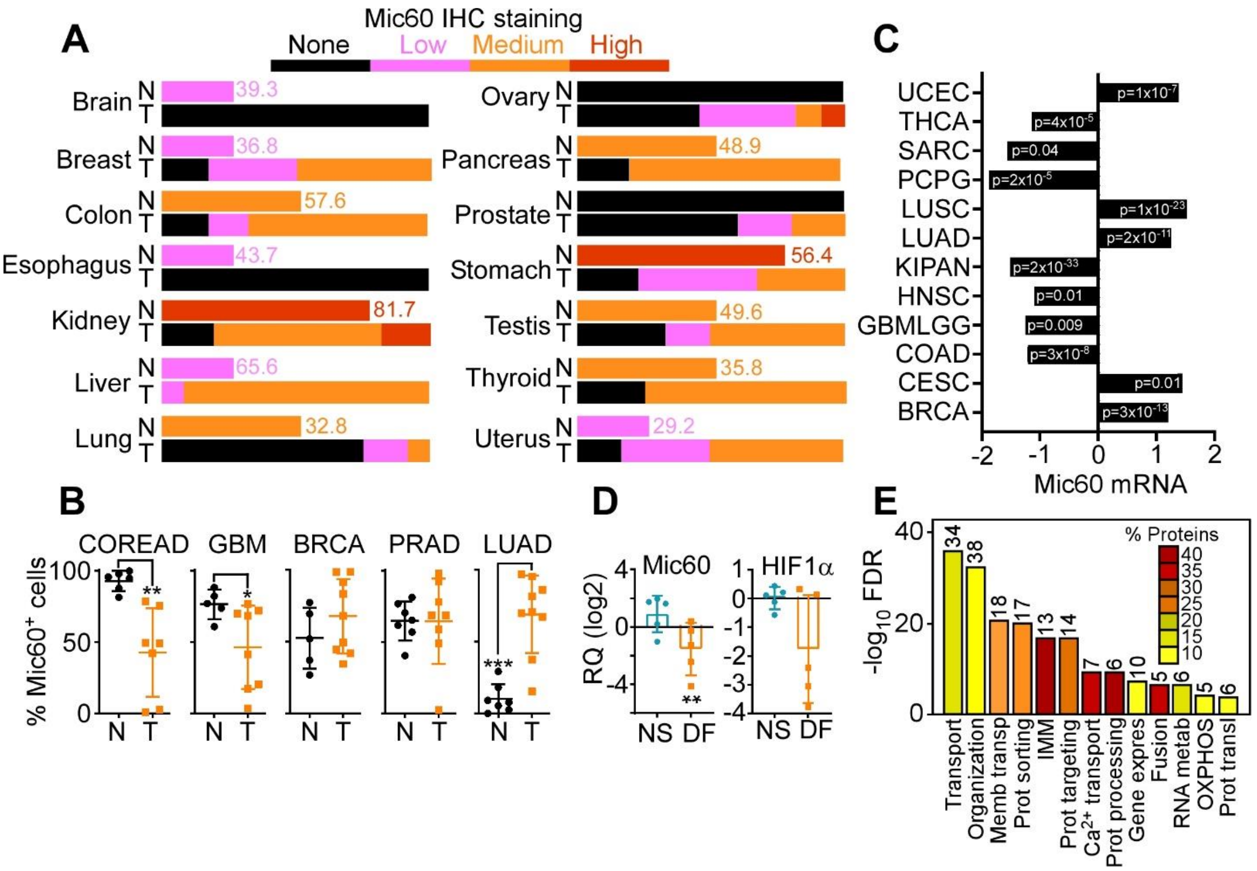

Fig. S1. Mic60 expression in cancer. (A) Mic60 expression by immunohistochemistry (IHC) in the Human Protein Atlas database. For each tissue, staining reactivity (None/Low/Medium/High) with antibody HPA036164 is indicated for normal (N) and the corresponding tumor (T) samples. Numbers correspond to consensus normalized expression values (nTPM). (B) Mic60 expression was examined by IHC in a universal tumor microarray and staining intensity in tumor (T) or adjacent normal (N) areas was quantified. Mean±SD (N=5-8 per condition). *, p=0.02; **, p=0.001 ***, p=0.0007. (C) Differential expression of Mic60 mRNA in tumor *vs*. normal tissues in The Cancer Genome Atlas (TCGA). Only statistically significant changes are shown. p values are indicated per each condition. (D) Patient derived GBM neurospheres (NS) were analyzed for changes in Mic60 (*left*) or HIF1α (*right*) mRNA expression by RT-PCR before or after differentiation (DF). Each symbol corresponds to an individual patient sample. Mean±SD (N=5).

**, $p=0.001$. (E) Ingenuity Pathway Analysis (FDR, <5%) of a Mic60 interactome identified by mass spectrometry in PC3 cells and comprising 119 proteins detected by at least 5 peptides with intensity >10-fold over controls. The number of proteins per each category are indicated.

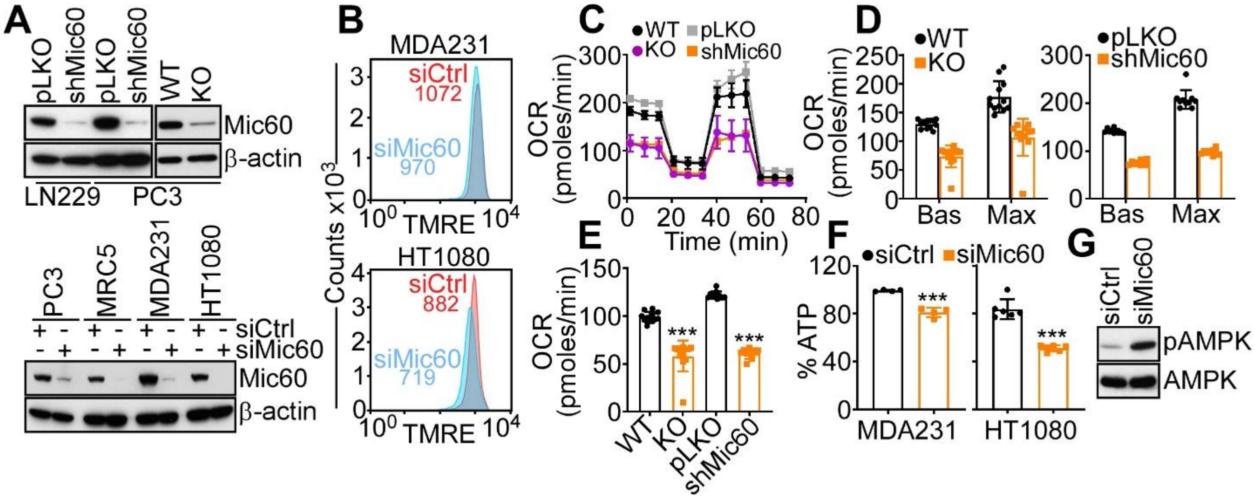

Fig. S2. Mic60 regulation of mitochondrial bioenergetics. (A) The indicated cell lines were stably transduced with pLKO or Mic60-directed shRNA (shMic60) (*top*), depleted of Mic60 by CRISPR/Cas9 (*top*) or transfected with control non-targeting siRNA (siCtrl) or Mic60-directed siRNA (siMic60) (*bottom*) and analyzed by Western blotting. WT, wild type; KO, knockout. (B) Breast adenocarcinoma MDA231 or osteosarcoma HT1080 cells were transfected as in (A, *bottom*) and analyzed for changes in mitochondrial inner membrane potential by TMRE staining and flow cytometry. Mean fluorescence intensities (MFI) per each condition are indicated. Representative experiment. (C) PC3 cells transduced with pLKO or shMic60 or WT or Mic60 KO PC3 (CRISPR/Cas9) cells were analyzed for oxygen consumption rates (OCR) on a Seahorse XFe96 Bioenergetics Flux Analyzer. Mean±SD (N=3). (D) The conditions are as in (C) and basal (Bas) and maximal (Max) respiration rates were quantified. Mean±SD (N=9-12). For all group comparisons, $p<0.0001$. (E) PC3 cells as in (C) were analyzed for rates of ATP production. Mean±SD (N=3). ***, $p<0.0001$. (F) MDA231 or HT1080 cells were transfected with siCtrl or siMic60 and analyzed for ATP production. Mean±SD. ***, $p<0.0001$. Representative experiment. (G) PC3 cells transfected with the indicated siRNA as in (F) were analyzed by Western blotting. p, phosphorylated.

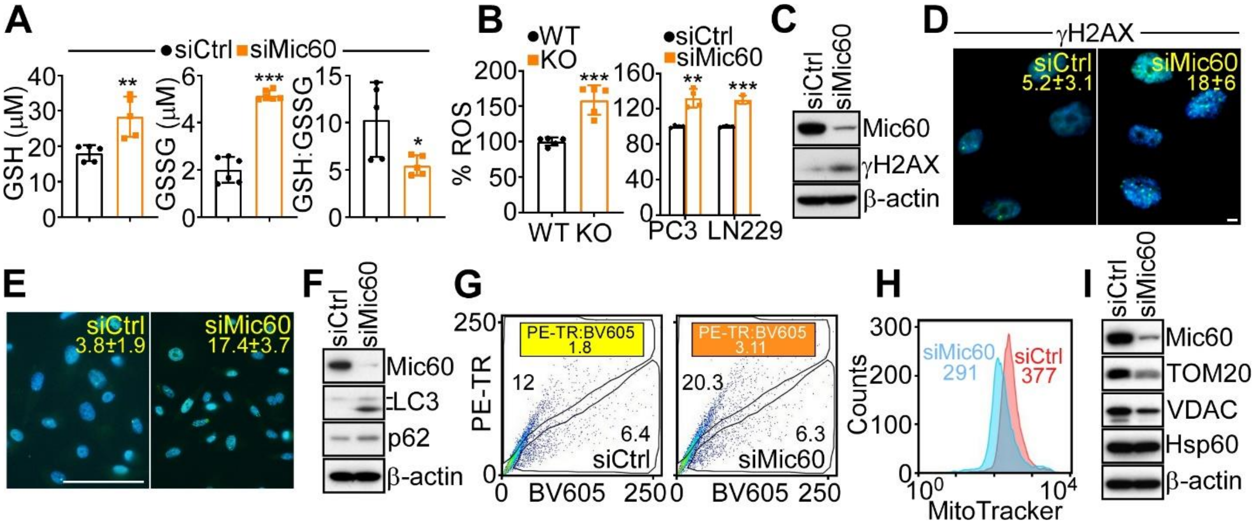

Fig. S3. Mic60 regulation of oxidative stress and mitochondrial quality control. (A) PC3 cells transfected with siCtrl or siMic60 were analyzed for GSH (*left*) oxidized GSH (GSSG, *middle*) or GSH:GSSG ratio (*right*). Mean±SD (N=3-4). *, p=0.02; ***, p<0.0001. (B) WT or Mic60 KO PC3 cells (*left*) or PC3 or LN229 cells transfected with siCtrl or siMic60 (*right*) were analyzed for cellular (*left*) or mitochondrial (*right*) ROS by CellRox or MitoSox staining and flow cytometry, respectively. Mean±SD (N=3). **, p=0.001; ***, p<0.0001-0.0003. (C and D) PC3 cells as in (A) were analyzed by Western blotting (C) and γH2AX subnuclear foci formation by fluorescence microscopy (D). Representative images. Scale bar, 10 μm. Mean±SD (N=14); p<0.0001. (E) PC3 cells expressing GFP-LC3 were transfected with siCtrl or siMic60 and analyzed by fluorescence microscopy. Representative images. Scale bar, 100 μm. The percentage of cells (mean±SD) with punctate GFP-LC3 staining is indicated (N=10). p<0.0001. (F) PC3 cells as in (A) were analyzed by Western blotting. (G) PC3 cells as in (A) expressing mitochondrial Keima-Red fluorescence reporter were analyzed by flow cytometry. The ratio phycoerythrin (PE)-Texas Red (TR)/BV605 staining is indicated. Representative tracings (N=2). (H) PC3 cells as in (A) were analyzed for mitochondrial mass by MitoTracker staining and flow

cytometry. Representative tracings. Mean fluorescence intensities (MFI) of a representative experiment are indicated. siCtrl, 1; siMic60, 0.73±0.04. Mean±SD (N=4), $p<0.001$. (H) PC3 cells as in (A) were analyzed by Western blotting.

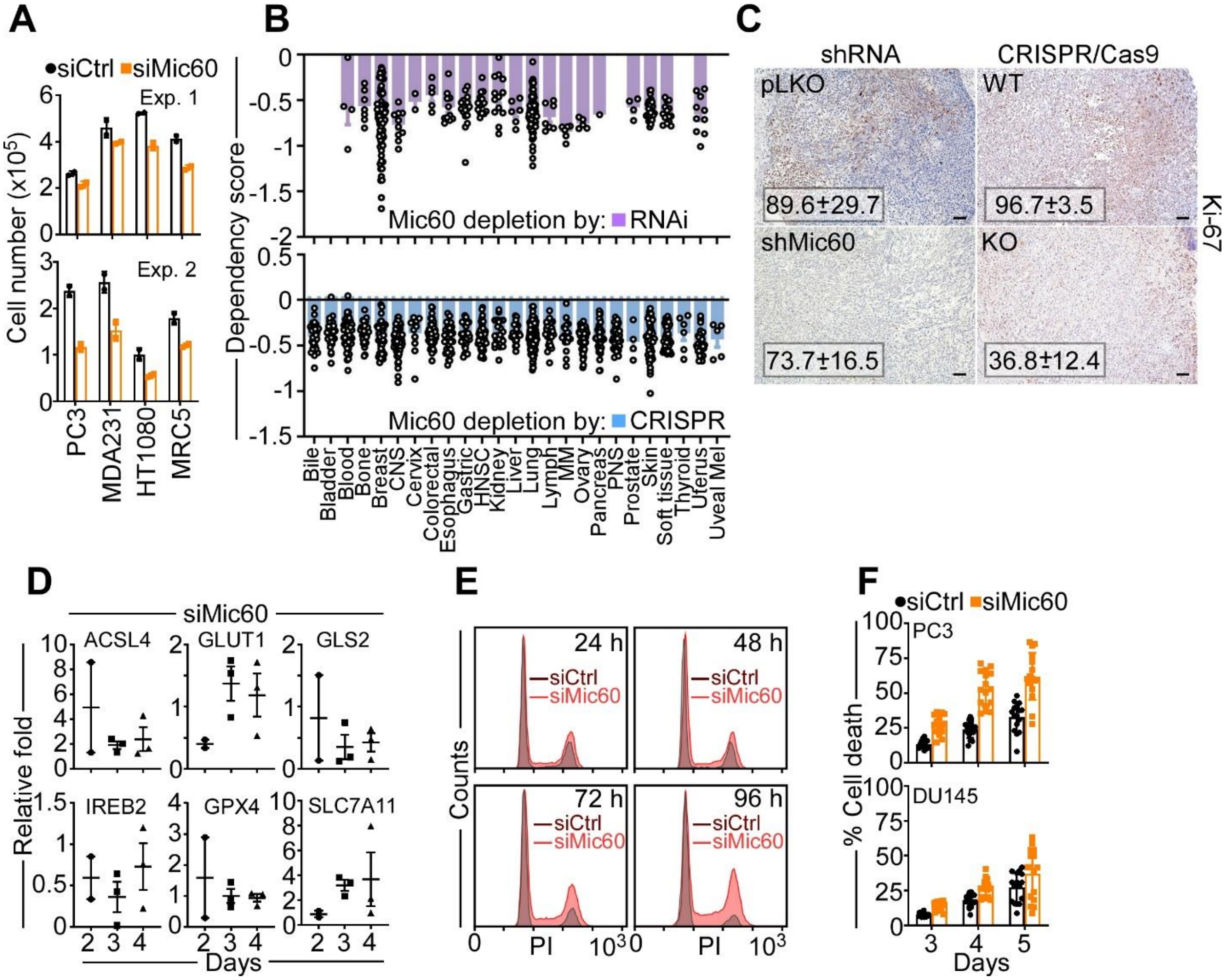

Fig. S4. Requirement of Mic60 for tumor cell proliferation. (A) The indicated tumor (PC3, MDA231, HT1080) or normal (MRC5) cell lines were transfected with siCtrl or siMic60 and analyzed for cell proliferation after 48 h by direct cell counting. Two independent experiments (Exp). Mean±SD. (B) Effect of Mic60 depletion by RNAi (*top*) or CRISPR/Cas9 (*bottom*) on tumor cell proliferation in the DepMap Project. The individual tumor types and a predicted dependency score per each condition are indicated. (C) PC3 cells transduced with pLKO or shMic60 or WT or Mic60 KO PC3 cells were injected s.c. in immunocompromised NSG mice and superficial tumors collected at d. 35 were stained with an antibody to Ki-67 and analyzed by IHC. Representative images. The percentage of Ki-67-positive cells (mean±SD) is indicated per

each condition (N=5-7). CRISPR/Cas9, $p<0.0001$. Scale bar, 300 μm. (D) PC3 cells transfected with siMic60 were analyzed for changes in mRNA expression of the indicated ferroptosis-associated genes at the indicated time intervals by RT-PCR. Mean±SEM (N=3). (E) PC3 cells transfected with siCtrl or siMic60 were stained with propidium iodide (PI) and analyzed by flow cytometry at the indicated time intervals (24-96 h). Representative histograms (N=4). (F) PC3 (*top*) or DU145 (*bottom*) cells transfected as in (E) were analyzed for cell death by trypan blue exclusion at the indicated time intervals. Mean±SD (N=12).

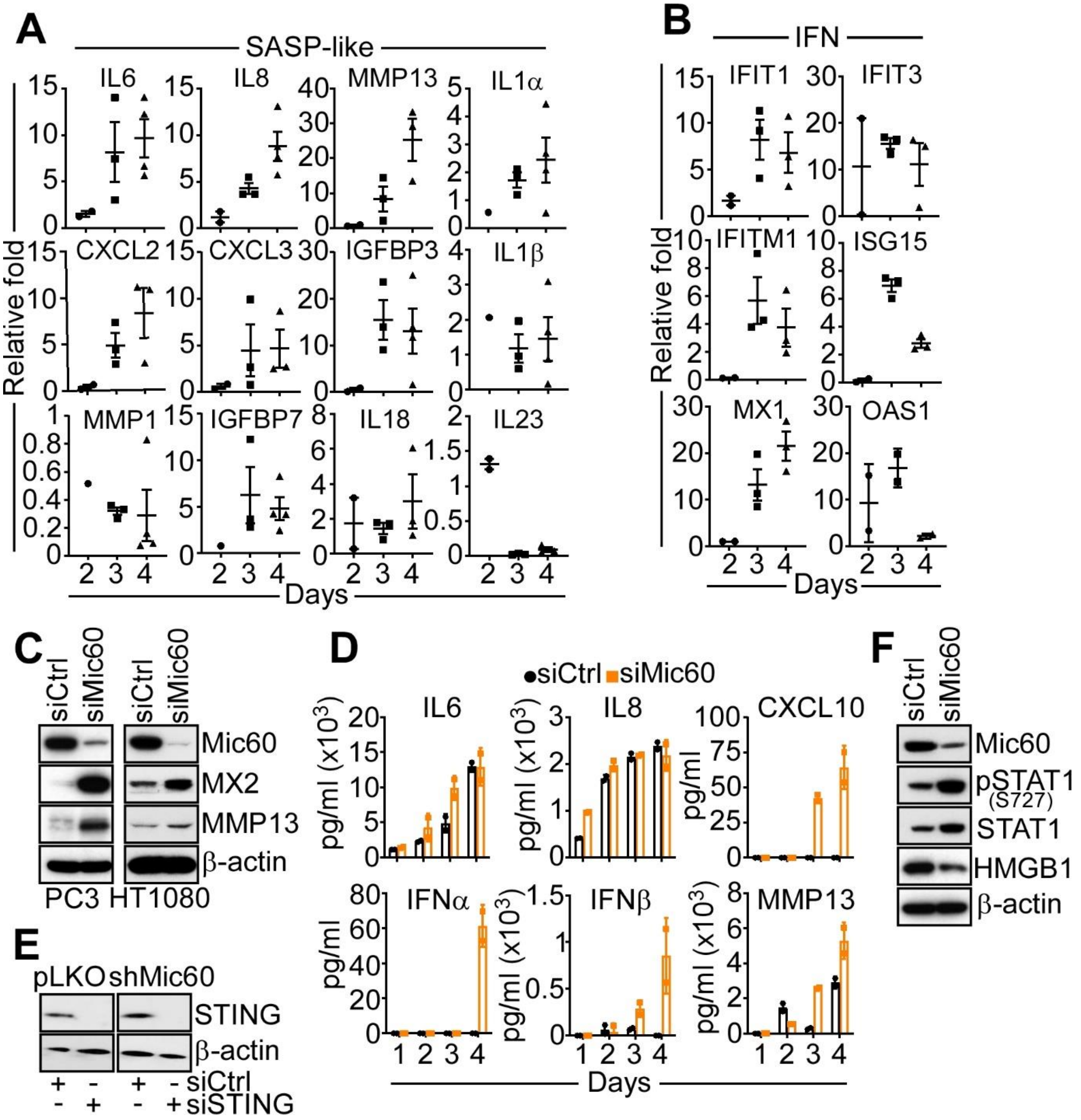

Fig. S5. Mic60 regulation of gene expression. (A and B) PC3 cells transfected with siMic60 were analyzed for mRNA expression of representative genes of SASP-like (A) or IFN (B) signaling by RT-PCR. Relative fold changes are indicated for each time interval tested (2-4 d). Median±SEM (N=3). (C) PC3 or HT1080 cells transfected with siCtrl or siMic60 were analyzed by Western blotting. (D) Conditioned media from PC3 cells transfected as in (A) were collected at the indicated time intervals (1-4 d) and analyzed for released cytokines/chemokines by ELISA.

Mean±SD (N=3). (E) PC3 cells expressing pLKO or shMic60 were transfected with siCtrl or STING-directed siRNA (siSTING) and analyzed by Western blotting. (F) PC3 cells transfected with siCtrl or siMic60 were analyzed by Western blotting.

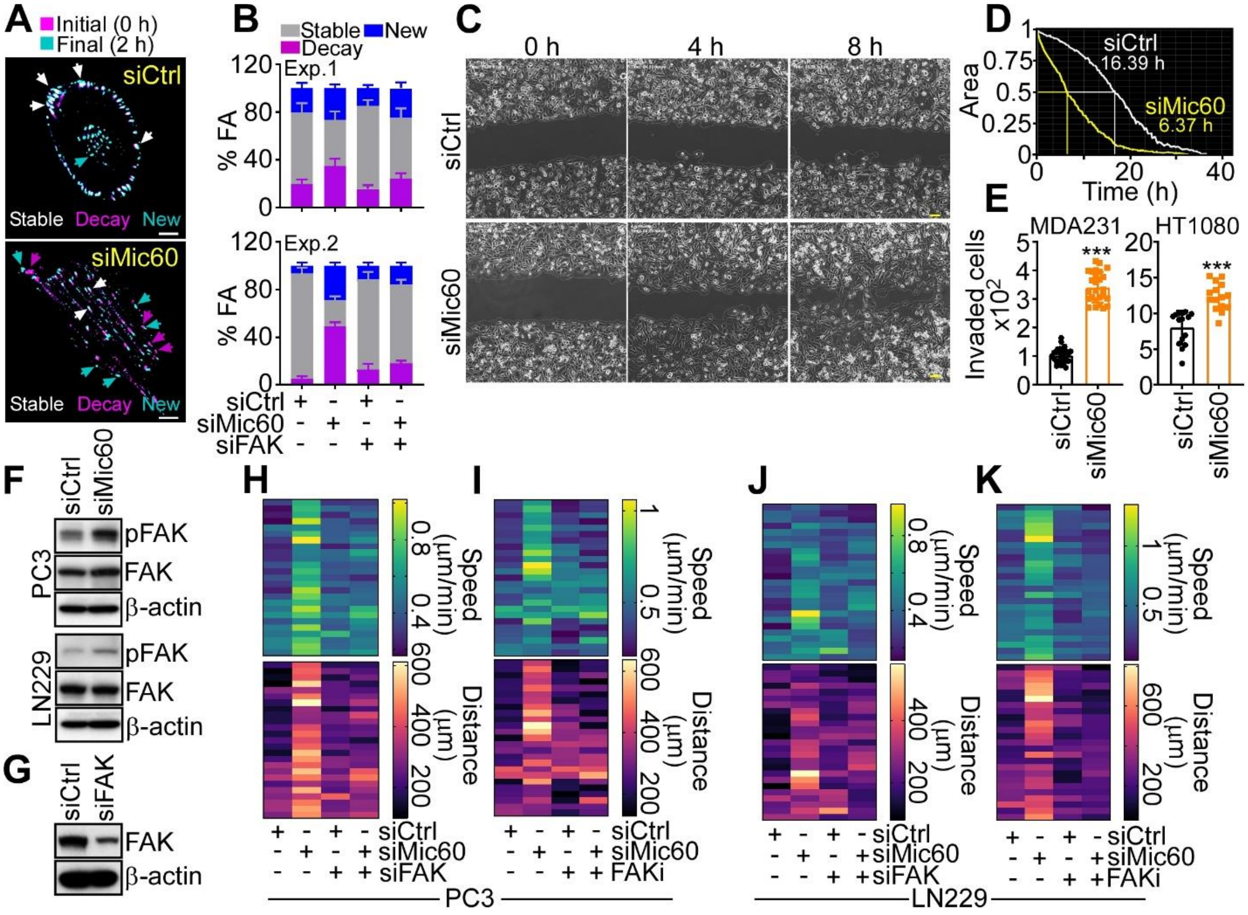

Fig. S6. Mic60 control of tumor cell motility. (A) PC3 cells transfected with siCtrl or siMic60 were labeled with Talin-RFP and analyzed for Focal Adhesion (FA) dynamics by time-lapse videomicroscopy. Representative merged frames at 0 h (magenta) and 2 h (cyan) are shown. Arrows, position of stable, decay and new FA. Scale bar, 10 μm. (B) The conditions are as in (A) and FA dynamics were quantified in the presence or absence of FAK-directed siRNA (siFAK). The percentage of new, stable or decay FA per each condition is indicated in two independent experiments (Exp). Mean±SD. (C and D) PC3 cells as in (A) were analyzed for directional cell migration in a wound closure assay at the indicated time intervals (C) and the half time (t½) (h) of wound closure was quantified (D). Representative images. Scale bar, 100 μm. (E) MDA231 or HT1080 cells were transfected as in (A) and analyzed for Matrigel invasion. Mean±SD. ***,

$p<0.0001$. (F) PC3 (*top*) or LN229 (*bottom*) cells as in (A) were analyzed by Western blotting. (G) PC3 cells transfected with siCtrl or siFAK were analyzed by Western blotting. (H and I) PC3 cells as in (A) were analyzed for single-cell motility in the presence of siCtrl or siFAK (H) or small molecule FAK inhibitor (FAKi, I) and the speed of cell movements and total distance traveled by individual cells were quantified in a heatmap (N=25). (J and K) The conditions are as in (H and I) except that LN229 cells were analyzed for single cell motility in the presence of siFAK (J) or FAKi (K) with quantification of speed of cell movements and total distance traveled by individual cells in a heatmap.

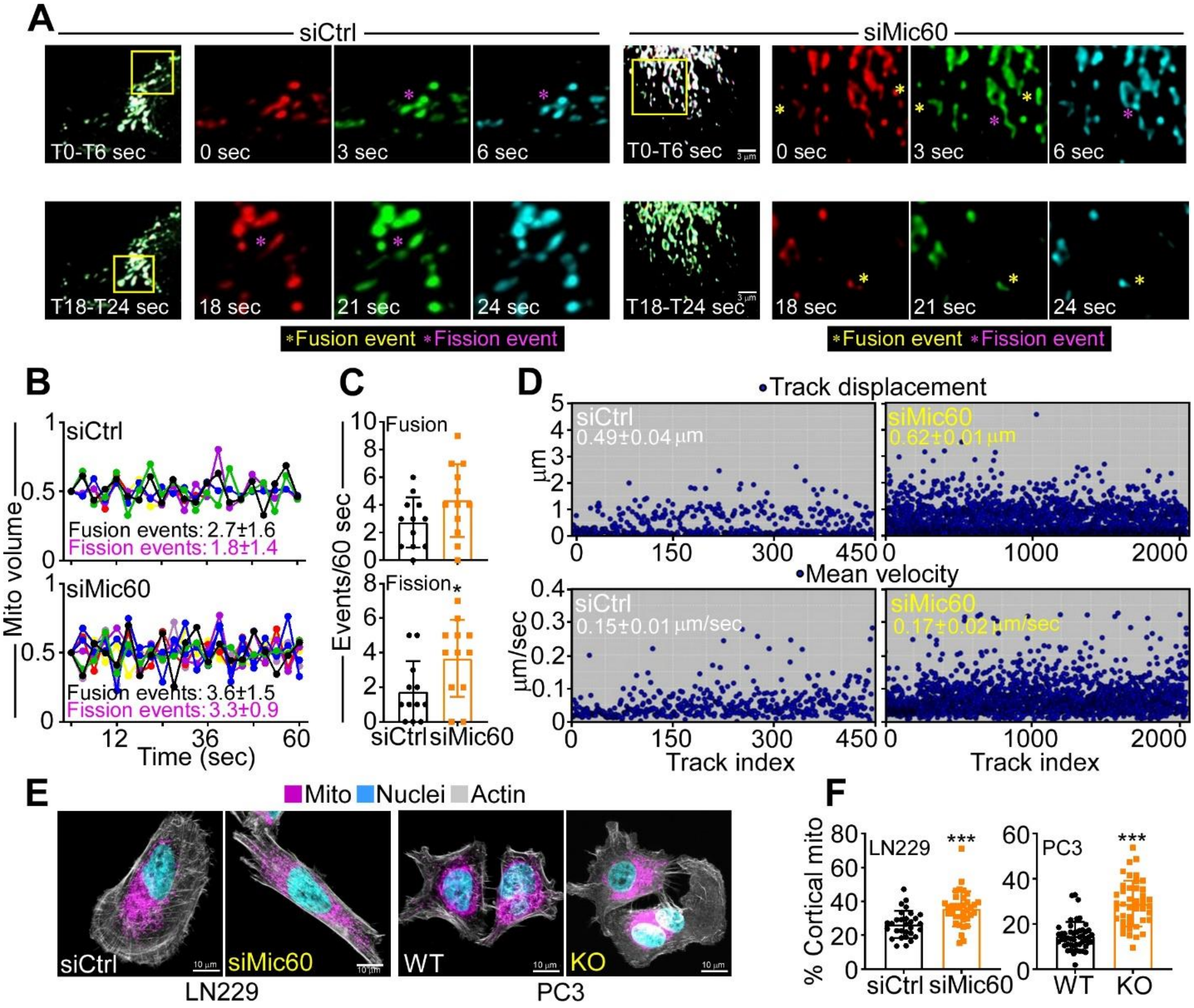

Fig. S7. Mic60 regulation of mitochondrial dynamics. (A) LN229 transfected with siCtrl or siMic60 were analyzed for mitochondrial dynamics by confocal microscopy. Images were acquired every 3 sec in live cells (*top*, time from 0 to 6 sec; *bottom*, time from 18 to 24 sec) and fusion and fission events were recorded during a 60-sec interval. *Yellow asterisk*, fusion event (fusion of 2 mitochondria in 1); *purple asterisk*, fission events (division of one mitochondrion in 2). Representative images. Scale bar, 3 µm. (B and C) The conditions are as in (A) and changes in mitochondrial (Mito) volume indicative of fusion (>1.5-fold volume) or fission (<1.5-fold volume) were analyzed over 60-sec intervals (B) and mitochondrial fusion (*top*) and fission

(*bottom*) events were quantified (C). Each line corresponds to an individual mitochondrion (N=6). Mean±SD (N=12). *, p=0.02. (D) PC3 cells as in (A) were analyzed for single mitochondrial motility by time-lapse videomicroscopy and distance traveled (*top*, track displacement) and mean mitochondrial velocity (*bottom*) were quantified. Each symbol corresponds to an individual mitochondrion. The average (mean±SD) of speed and distance traveled is indicated. Representative experiment (N=3). (E and F) LN229 cells as in (A) (*left*) or WT or Mic60 KO PC3 cells (*right*) were analyzed by confocal microscopy (E, representative images) and mitochondrial redistribution to the cortical cytoskeleton was quantified per each condition tested (F). Scale bar, 10 μm. Mean±SD (LN229, N=30-36; PC3, N=42-48). ***, p=0.0002-<0.0001.

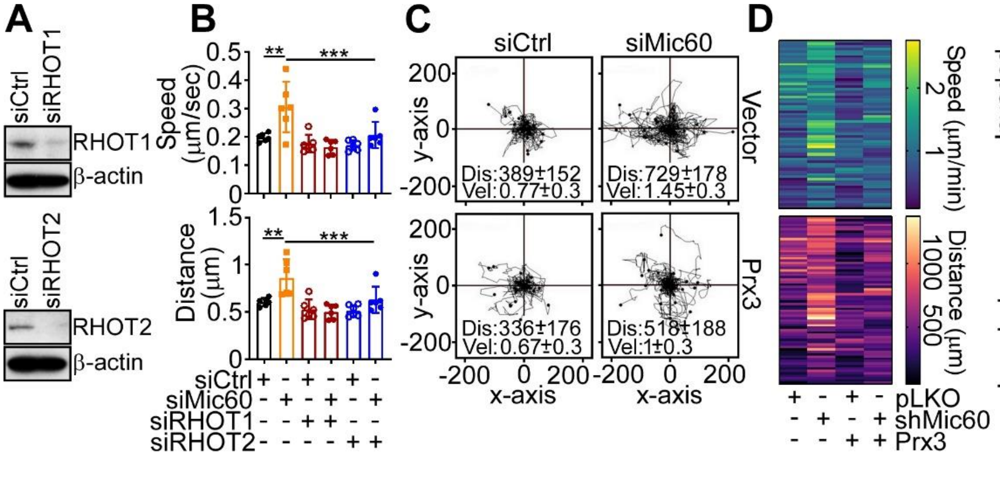

Fig. S8. Requirements of Mic60 regulation of mitochondrial-fueled tumor cell motility. (A) LN229 cells transfected with siRNA to RHOT1 (*top*) or RHOT2 (*bottom*) were analyzed by Western blotting. (B) LN229 cells as in (A) were transfected with siCtrl or siMic60 and analyzed for mitochondrial motility by time-lapse videomicroscopy with quantification of mitochondrial speed (*top*) or distance traveled (*bottom*) by individual mitochondria. Mean±SD (N=6). Speed, **, p=0.003; ***, p=0.0002-0.0004; distance, **, p=0.006; ***, p=0.0001-0.0003. (C) PC3 cells transfected with siCtrl or siMic60 were reconstituted with vector or Prx3 and analyzed for single-cell motility in 2D contour plots by time-lapse videomicroscopy. Each line corresponds to the movements of an individual cell. The average (mean±SD) speed of cell movements (Velocity, Vel, μm/min) and total distance traveled by individual cells (Distance, Dis, μm) are indicated. Representative experiment. (D) PC3 cells transduced with pLKO or shMic60 were reconstituted with Prx3 and the speed of cell movements (*top*) and total distance traveled by individual cells (*bottom*) was quantified in a heatmap. Speed (μm/min), pLKO, 0.81±0.44; shMic60, 1.3±0.54; shMic60+Prx3, 0.94±0.36, distance traveled (μm), pLKO, 442±220; shMic60, 682.8±271.6; shMic60+Prx3, 473.4±183.6. Mean±SD (N=70-89). (E) PC3 cells reconstituted as in (D) were analyzed for Matrigel invasion in a Boyden chamber. Mean±SD (N=38-46). ***, p<0.0001-

0.0006; ns, not significant. (F) PC3 cells transfected with siCtrl or siMic60 were incubated with mitochondrial superoxide scavenger, MitoTempo (MT) and analyzed for Matrigel invasion. Mean±SD (N=2). ***, $p<0.0001$; ns, not significant.

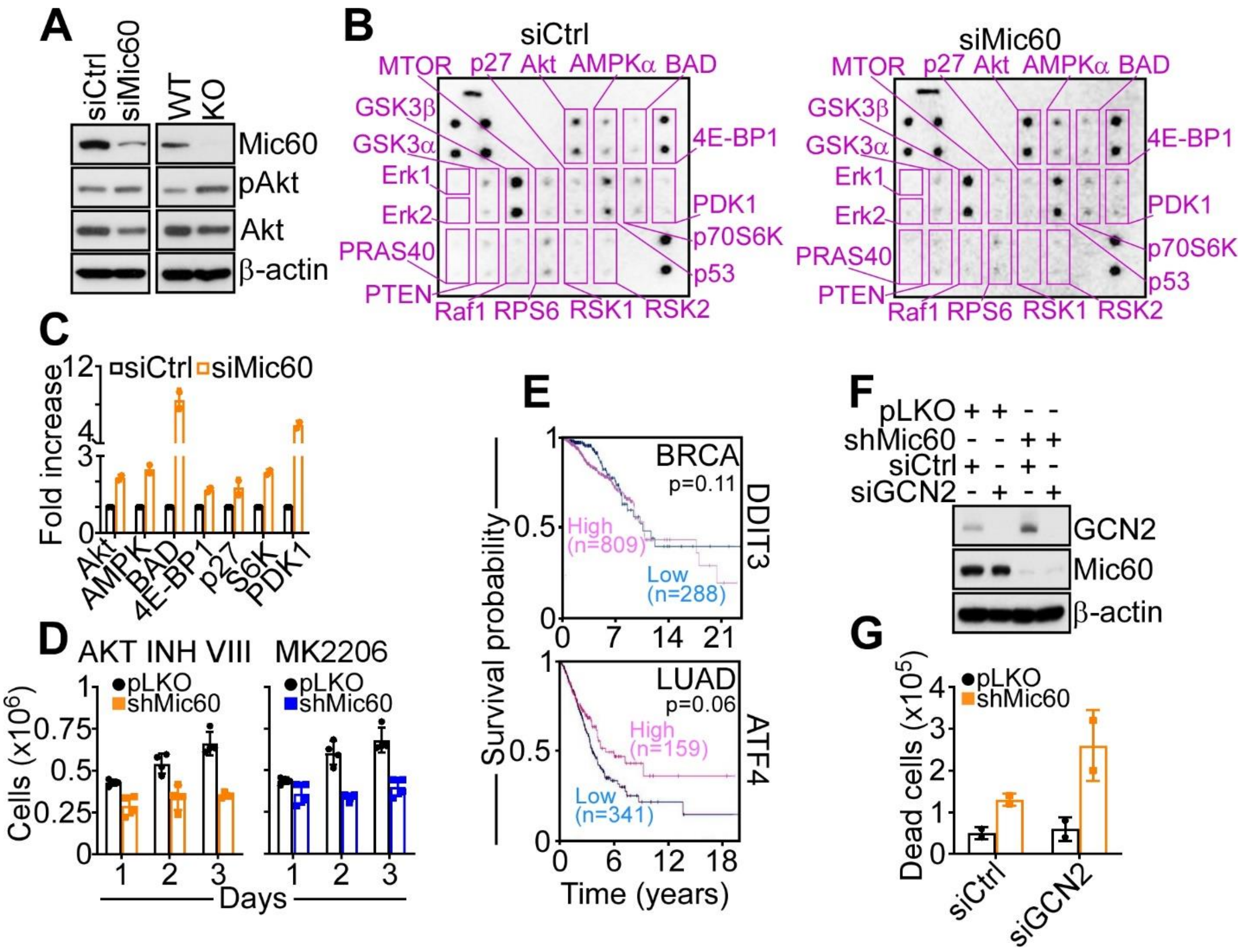

Fig. S9. Activation of adaptive GCN2/Akt signaling in *Mic60-low* tumors. (A) PC3 cells transfected with siCtrl or siMic60 (*left*) or WT or Mic60 KO PC3 cells (*right*) were analyzed by Western blotting. p, phosphorylated. (B and C) PC3 cells transfected with siCtrl or siMic60 were analyzed in an Akt pathway phospho-array (B) and the fold-increase in signal intensity in Mic60-depleted cells normalized to control cultures was quantified by densitometry (C). The position of the individual proteins in the phospho-array is indicated. Representative experiment. Mean±SD (N=2). (D) PC3 cells transduced with pLKO or shMic60 were incubated with small molecule Akt inhibitor VIII (*left*) or MK2206 (*right*) and analyzed for cell proliferation at the indicated time intervals by direct cell counting. Mean±SD (N=3). (E) Kaplan-Meier survival curves for differential expression of DDIT3 (*top*) or ATF4 (*bottom*) in *Mic60-high* tumors BRCA or LUAD

in the Human Protein Atlas database. The number of patients per each condition and corresponding p values are indicated. (F) PC3 cells as in (D) were transfected with siCtrl or GCN2-directed siRNA (siGCN2) and analyzed by Western blotting. (G) The conditions are as in (F) and transfected PC3 cells were analyzed for cell death by Trypan blue exclusion and light microscopy after 72 h. Mean±SD.
